# Simulating direct shear tests with the Bullet physics library: A validation study

**DOI:** 10.1371/journal.pone.0195073

**Published:** 2018-04-19

**Authors:** Ehsan Izadi, Adam Bezuijen

**Affiliations:** 1 Laboratory of Geotechnics, Ghent University, Ghent, East Flanders, Belgium; 2 Deltares, 2600 MH Delft, The Netherlands; University of Naples Federico II, ITALY

## Abstract

This study focuses on the possible uses of physics engines, and more specifically the Bullet physics library, to simulate granular systems. Physics engines are employed extensively in the video gaming, animation and movie industries to create physically plausible scenes. They are designed to deliver a fast, stable, and optimal simulation of certain systems such as rigid bodies, soft bodies and fluids. This study focuses exclusively on simulating granular media in the context of rigid body dynamics with the Bullet physics library. The first step was to validate the results of the simulations of direct shear testing on uniform-sized metal beads on the basis of laboratory experiments. The difference in the average angle of mobilized frictions was found to be only 1.0°. In addition, a very close match was found between dilatancy in the laboratory samples and in the simulations. A comprehensive study was then conducted to determine the failure and post-failure mechanism. We conclude with the presentation of a simulation of a direct shear test on real soil which demonstrated that Bullet has all the capabilities needed to be used as software for simulating granular systems.

## Introduction

Physics engines are extensively used in the video gaming, animation and movie industries to produce physically plausible scenes. This allows game makers, animators and movie editors to dispense with key frames for manually setting the movements of animated objects. Depending on the purpose for which they are developed and the level of development, physics engines can simulate certain physical systems such as rigid bodies, soft bodies and fluids (or combinations of these systems). The principal approach underlying each simulation of a system can vary depending on the physics engine type. For instance, hard contact or soft contact methods can be adopted for rigid-body simulation; finite element or position-based methods are used are used for soft-body simulation; and smoothed particle hydrodynamic approach has become very popular for fluid simulation in recent years and it has been employed in some physics engines [1, 2]. The inclusion of all these capabilities in a single software package makes it a versatile, fast and powerful tool for the simulation of a wide range of engineering-related problems, especially in geotechnical engineering. Furthermore, physics engines are developed mainly for gaming and animation purposes and so the source codes are written so that they are stable, optimized and fast: Bullet, PhysX and Havok are amongst the physics engines that can share the computational load on a graphics processing unit (GPU) in order to shorten calculation times [3–5]. Perhaps one of the best examples of the efficiency assessment of physics engine is the study of Hamano et al. [6]. Within the framework of simulation of collapsing structures, they assessed and compared the efficiency of three major physics engines, namely PhysX [5], Bullet Physics Library [3], and Open Dynamics Engine (ODE) [7] on four computers equipped with different set of CPU and GPU settings. In their simulations several rigid bodies and constraints (representing the elements of structures) were included. They compared the processing time of the investigated physics engines regarding various iteration counts, number of rigid bodies as well as the number of constrained rigid bodies. Giris and Wells [8] investigated the time spent on various GPU-based computational operations by using a simple 2D rigid body simulator on GPU. They showed that more than 75% of the processing time is spent on the collision detection amongst rigid body parts.

Although physics engines were mainly developed for the game-related industries, they are being employed in different fields of science and technology, with robotics being the field that has probably used physics engines most [9–11]. NASA used Bullet to develop a tensegrity-specific simulator for tensegrity robots [12]. Google has been using Bullet physics engine to simulate robots [3]. Bullet is also used in the virtual reality for on-orbit servicing project [13] for the real-time simulation of the realistic dynamic and kinematic behaviour of satellite (and robot) components for various on-orbit servicing tasks. As opposed to games, where it is usually enough for results to just look plausible, high levels of accuracy are crucial in projects of this kind. In addition, physics engines have been used to study the stability of cranes [14] and the behaviour of bridges when subjected to collapse [15].

**Fig 1:**
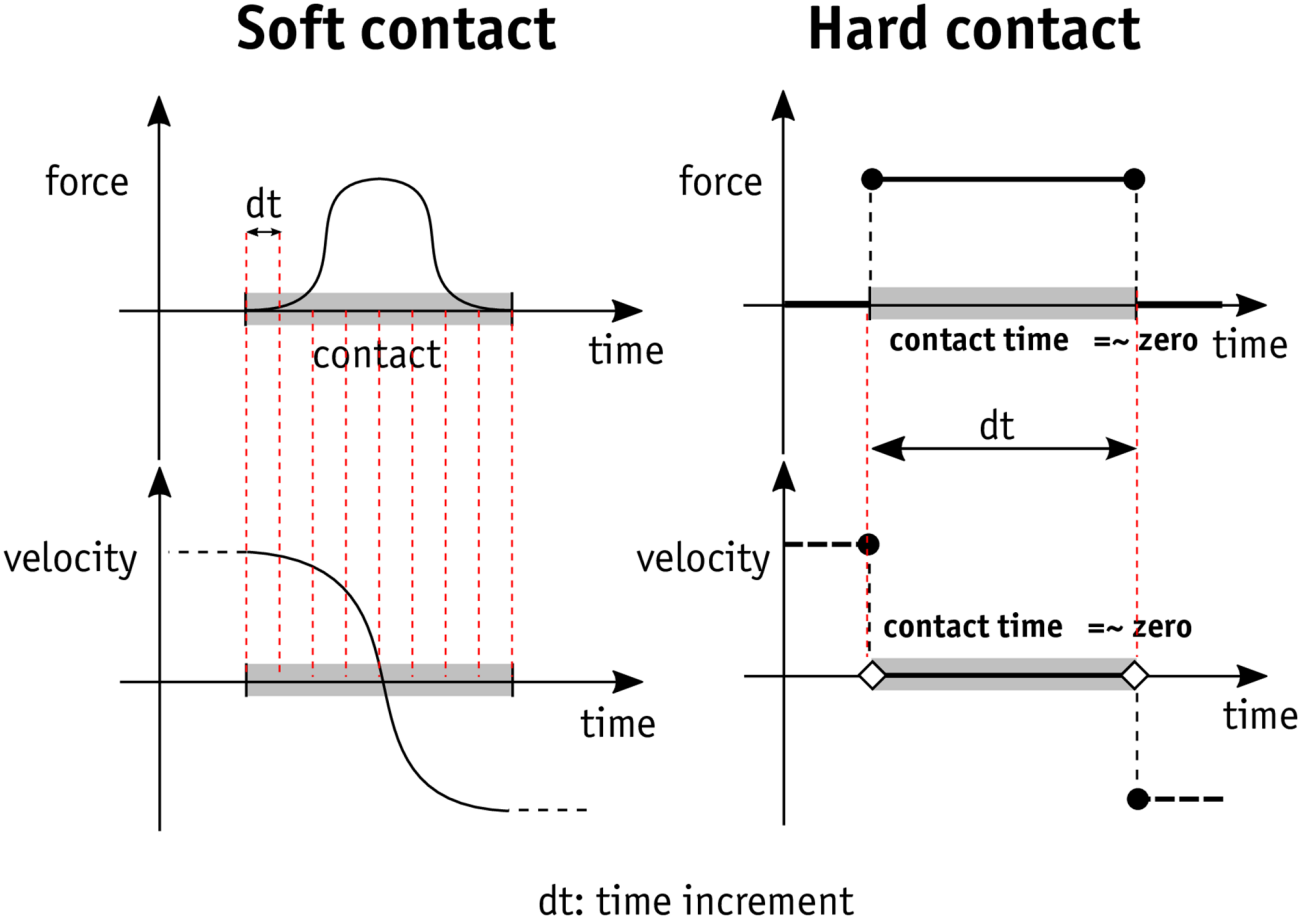
Soft-contact vs hard-contact approach.

In geotechnical engineering, the best way of capturing the real response of granular systems at the micro- and meso-scales is probably to use discrete element methods. The discrete element method (DEM) was introduced by Cundall and Strack in 1979 [16] and it is used by many researchers [17–19]. The emergence of DEM in granular simulations has been successful and promising. Although DEM needs more computational power and therefore results in slower calculations than continuum-based methods, it has overcome the drawbacks of continuum-based approaches. For instance, with very simple contact laws, DEM can capture the complex behaviour of particulate media (such as stress hardening or softening and dilation) [20]. DEM has become very popular with researchers since many key complex mechanical responses that are specific to soil and other granular materials can be captured with a large degree of confidence [17]. Contact dynamics (CD) is a discrete-based method that is used for the rigid-body simulation modules in physics engines There is a subtle difference between DEM and CD that corresponds to how contact resolution is considered. In DEM and CD, contacts are handled using soft and hard contact approaches respectively. This can be illustrated by assuming that two objects are colliding at a certain velocity. The contact force-time and velocity-time graphs for both approaches are shown in Fig 1. As becomes clear from Fig 1, the soft contact approach allows objects to overlap and the magnitude of normal and frictional forces at contacts depends on the amount of overlap between objects. In other words, there is a penetration-dependent scale for the derivation of the contact forces. This implies that the contact needs to be solved in increments of discrete time, compelling the solver to work with relatively small time increments. By contrast, the contact itself is of no interest in the hard contact concept and it is not modelled explicitly. Instead, the effect of the contact is taken into account in the post-collision movement of objects as a result of momentum exchange [17, 21]. In hard contact approaches, then, the time increment selected can be larger than in soft contacts. This reduces calculation times, especially when simulating highly dynamic regimes in granular systems. Lim et al. [22] look at the differences between DEM and CD in greater detail.

Hard contact methods were not widely used in granular simulation until recently [23–31]. This could be due to the fact that implementation of the solver algorithm of hard contact approach is more difficult than that of soft contact approach, despite the fact that the time increments in the hard contact methods make it computationally cheaper than the soft contact approach [32]. Initially, the CD method was proposed and developed by Moreau and Jean [26, 33, 34] and many researchers then continued to use it in the field of granular simulation.

Given the above, it was decided to explore the idea of using physics engines to simulate particulate media. Nevertheless, the accuracy of these engines is still a matter of debate since engine accuracy has often been sacrificed by developers to make calculations faster. The emergence of physics engines in the field of granular simulation is very new and there have been very few studies using them. Izadi and Bezuijen [35] and Pytlos et al. [36] have taken steps in this area. Izadi and Bezuijen [35] performed a series of pluviation and vibration simulations on poorly graded gravel using the Bullet physics engine in 3D and validated their results on the basis of laboratory experiments. The study was important in two respects: first, it looked at the modelling of gravel geometries by Vorono-shattered polyhedrons. The effect of the shape of particles was therefore taken into account, although the same packing configuration as in the laboratory samples was not generated in their study. Secondly, their simulations included highly dynamic regimes. Pytlos et al. [36] conducted a 2D simulation of an angle of repose test as well as a retaining wall test on cohesionless particles made of randomly-shaped polygons. They concluded that the physics engine can be used to simulate the behaviour of frictional soils. In another study, Pytlos et al. [37] showed that the critical state soil behaviour can be captured using a BOX 2D physics engine [38] in a 2D numerical study.

Recent studies have not validated the engines in terms of quantitative engineering values such as stresses and strains. A validation study is therefore needed to use the software confidently in research and practice. The main aim of the present study was to validate Bullet at an advanced level. A series of direct shear tests were therefore carried out on metal precision beads with a controlled packing configuration. This type of configuration makes it possible to replicate identical samples in the simulations. A comprehensive study was then conducted to identify the failure and post-failure mechanism in the sample and, finally, a simulation of real soil using irregularly-shaped polyhedrons in a direct shear test was conducted to determine whether the soil behaviour was realistic.

## Bullet physics library

The Bullet physics library was selected for this study because Bullet is free, open source and cross-platform software, making it very simple to work with on any platform. It also allows the user to share the computational load on a GPU, which makes the simulation up to 30 times faster than on a central processing unit (CPU) [39]. Bullet includes many features that makes it easy to use in ways tailored to the requirements of the user and, finally, it is being rapidly improved by a growing community of users and developers [3]. A few attempts have been made to establish benchmark tests for different physics engines [40, 41]. However, those studies do meet engineering requirements and the parameters were set on the basis of values that satisfy game developers rather than engineers. Furthermore, since most of physics engines are undergoing rapid development, the benchmarks can be outdated even before publication. In this study, daily builds of Bullet were used to take advantage of new developments.

Bullet uses CD as the main approach for solving rigid-body systems. Like other DEM software [42, 43] and other physics engines [4, 5], Bullet uses a time-stepping scheme to solve rigid-body systems. First, Bullet runs a collision-detection procedure to identify the colliding bodies. Then, relying on the velocities of the objects before the collision, it calculates the velocities after collision and, finally, updates the new velocities, positions and orientations of the rigid bodies based on the Newton-Euler laws of motion. The CD fundamentals are discussed briefly below.

**Fig 2:**
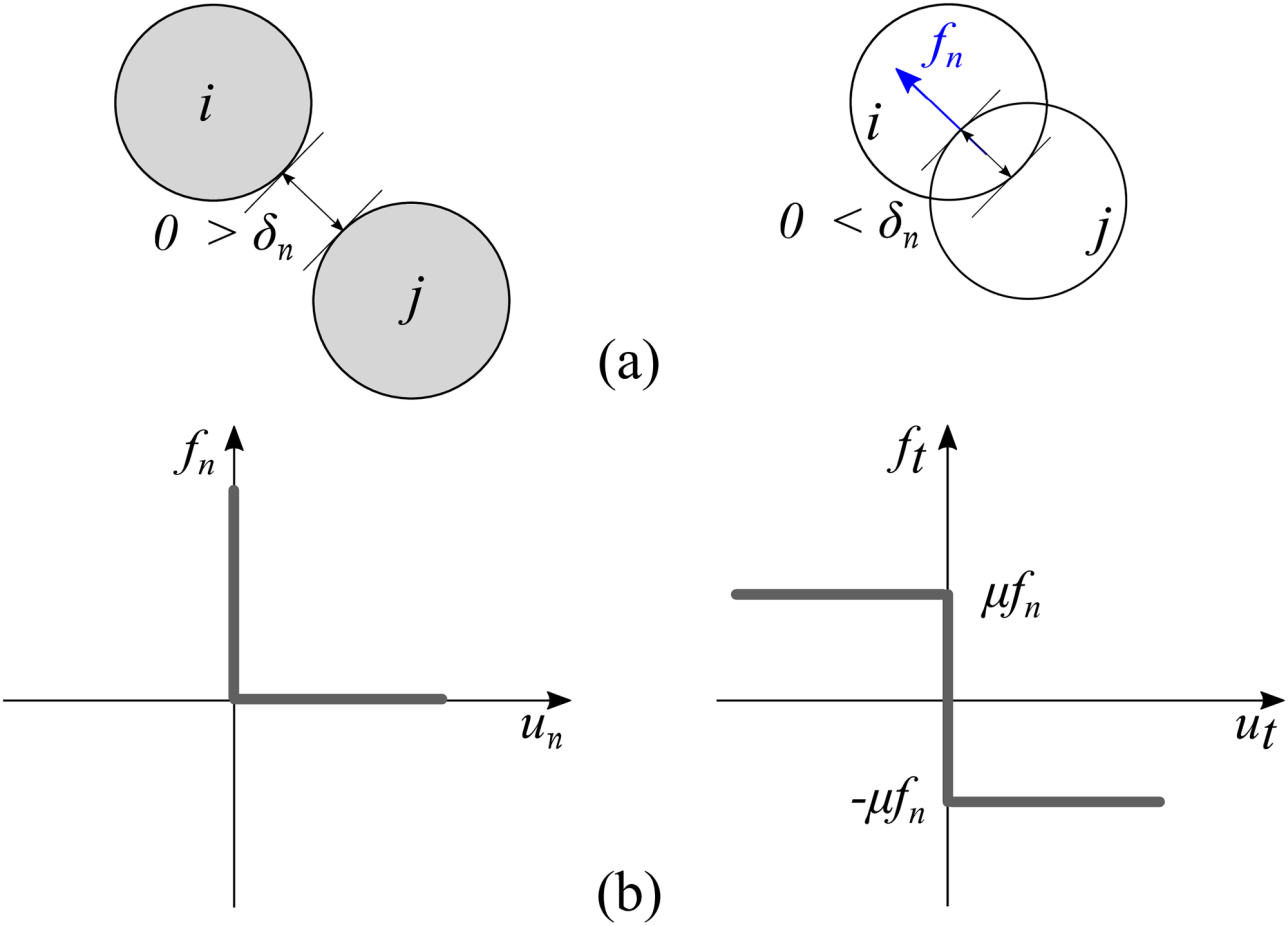
Sample contact between two objects (a) and the corresponding Signorini’s complementarity relation (b).

Let us assume that there is a contact at a specific time during a simulation between two objects, *i* and *j*. As long as the distance between the boundaries of the two objects, *δ*_*n*_, remains negative, there is no contact force between the objects (Fig 2(a)). However, as soon as *δ*_*n*_ ≥ 0, a force is mobilized at the contact point. Depending on the velocity of the colliding objects, it is possible to define the following Signorini conditions [33]:

$$\{\begin{array}{l} u_{n}\geq 0\to f_{n}=0\\ u_{n}=0\to f_{n}\geq 0 \end{array}$$ (1)

Where $u_{n}=\dot{\delta_{n}}$ is the normal component of contact velocity (the relative velocity of two objects) and *f*_*n*_ is the normal component of contact force, as illustrated in Fig 2(b). Eq 1 results in a problem where either one variable is positive and the other is zero or vice-versa. To formulate the tangential forces in the contact, Signorini’s complementarity problem can be defined again:

$$\{\begin{array}{l} u_{t}>0\to f_{t}=-\mu f_{n}\\ u_{t}=0\to f_{t}=-\mu f_{n}\leq f_{t}\leq \mu f_{n}\\ u_{t}<0\to f_{t}=\mu f_{n} \end{array}$$ (2)

Where *u*_*t*_ and *f*_*t*_ are the tangential component of the velocity and force of the contact respectively. The graph for this complementarity equation is shown on the right-hand side of Fig 2(b). As stated above, CD handles the contacts in such a way that the contact itself is not of interest and the effect of contact is derived from the pre-collision state to determine the post-collision state. It is therefore convenient to split the contact velocities into left-limit velocity, *u*^−^, and right-limit velocity, *u*^+^, which correspond to the velocities at the times of *t*_0_ and *t*_0_ + *δ*_*t*_ respectively. Assuming that *u*_*n*_ and *u*_*t*_ are the weighted means for the left-hand and right-hand contact velocities, one can rewrite the velocities as:

$$\begin{array}{c} u_{n}=\frac{u_{n}^{+}+e_{n}u_{n}^{-}}{1+e_{n}} \end{array}$$ (3)

$$\begin{array}{c} u_{t}=\frac{u_{t}^{+}+e_{t}u_{t}^{-}}{1+e_{t}} \end{array}$$ (4)

Where *e*_*n*_ and *e*_*t*_ are the normal and tangential coefficients of restitution respectively. In the CD approach, the contact law is formulated in such a way that the energy inside the system is damped in two ways. In the first, energy is lost due to the incomplete resilient behaviour of objects when colliding, which becomes important in dynamic regimes. The second is determined by the local movements of objects relative to each other. The value for the coefficient of restitution and friction can therefore have a profound influence on granular behaviour.

**Fig 3:**
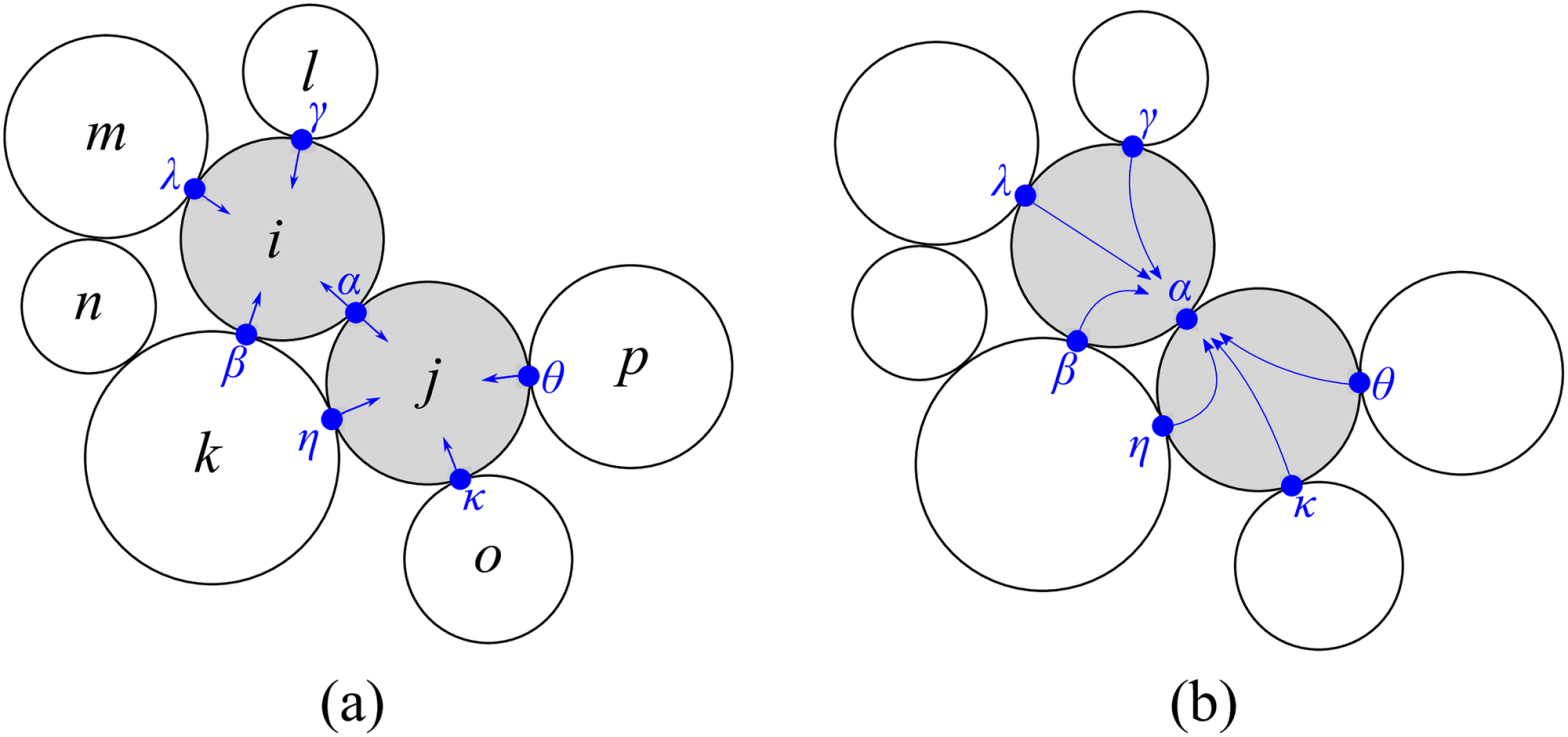
A cluster of objects in contact: Schematic diagram of the contact forces (a); and the nested relation between the contact forces (b).

This method makes it possible to write the equations of motion for each particle. Considering a system of particles in two dimensions as shown in Fig 3(a), if the calculation of the contact force at the contact *α* is of interest, equations of motion for neighbouring particles can be written:

$$\begin{array}{c} \begin{array}{c} m\;\dot{\vec{U}}\;=\;\vec{F}\;+\;{\vec{F}}_{ext}\\ I\;\dot{\omega }\;=\;M\;+\;M_{ext} \end{array} \end{array}$$ (5)

Where *m*, *I*, $\dot{U}$ and $\dot{\omega }$ are mass, moment of inertia, rate of velocity change and rate of angular velocity change respectively. $\vec{F}$ and *M* are the resultant of the forces and moments imposed by all the contacts that each particle can have and, likewise, ${\vec{F}}_{ext}$ and *M*_*ext*_ are the resultants of external forces and moments on the particle of interest. In this example, for particle *i*, $\vec{F}=\sum_{k=\alpha }^{\beta ,\lambda ,\gamma }f^{k}$ and $\vec{M}=\sum_{k=\alpha }^{\beta ,\lambda ,\gamma }M^{k}$ where *β*, *λ* and *γ* are the neighbouring contacts that influence the contact *α*. Solving the problem in a discrete time-stepping scheme allows us to replace $\dot{U}$ by $\frac{U^{+}-U^{-}}{\delta_{t}}$ and $\dot{\omega }$ by $\frac{\omega^{+}-\omega^{-}}{\delta_{t}}$. Since the aim is to determine the contact force for the contact of interest, here $\vec{f^{\alpha }}$, it is necessary to derive equations of motion for the contacts from the equations for the motion of particles. Some conversions are therefore needed:

$$\begin{array}{c} u\;=\;G\;U \end{array}$$ (6)

$$\begin{array}{c} F\;=\;G^{T}\;f \end{array}$$ (7)

Where *G* is a matrix containing the information about the configuration of the neighbouring particles that is updated for each time step. This conversion eventually allows for the derivation of the following relationship:

$$\begin{array}{c} \vec{f^{\alpha }}\;=\;{\vec{F}}_{network}\;+\;{\vec{F}}_{restitution}\;+\;{\vec{F}}_{ext}\; \end{array}$$ (8)

The left-hand side of Eq 8 corresponds to the contact forces (in the normal and tangential directions) of interest and, in the right-hand side composed of ${\vec{F}}_{network}$, the forces come from the neighbouring contacts (contacts between particles *i* and *j*): ${\vec{F}}_{restitution}$, which is the force due to the left-hand velocity of the contact, which is always dynamic in nature, and ${\vec{F}}_{ext}$, which consists of other forces imposed on the contact between neighbouring particles (*i* and *j*) such as gravity forces, boundary forces, etc. As can be seen, there is a nested relationship here between the neighbouring contact forces (Fig 3(b)) of the neighbouring particles. A denser configuration therefore results in a more complex problem. Moreover, Eq 8 reveals a unique feature of CD, which is that it brings together two different conditions that may occur in a granular system: one is the collisional state characterized by binary shocks and incomplete energy restitution and the other is the static state involving multiple contacts and force balances. A linear complementarity problem of this kind can be solved using a wide variety of methods. In Bullet, the standard solver—the ‘sequential impulse constraint solver’—uses the iterative projected Gauss Seidel approach and we use that solver in the present paper.

The approach presented is simply a summary on how CD method solves rigid body systems. For further details, readers are referred to the work of Radjai and Richefeu [27], which provides an excellent description of the approach.

## Experimental programme

The proper selection of the testing material is of the utmost importance for the purposes of the validation study. Given one of the basic assumptions of CD method, which does not allow objects to overlap, the material must be very stiff and elastic deformation is therefore negligible. Furthermore, the geometry of the grains should preferably be as idealized as possible in order to keep the modelling procedure in Bullet as straightforward as possible. In addition, the surface of the selected material should be uniform and the tolerances in quality control must be limited. A comprehensive search was conducted to select suitable material for the tests and the grade 25 chrome steel precision beads were selected for this study. According to the information provided by the manufacturer (Thompson Precision Co.), the tolerance for both bead diameter and sphericity is ±0.6*μ*m. Given the size of the shear box available (60×60 mm), diameter beads measuring 4 mm were selected. These beads can manage loads of up to ≃780 kg before cracking. Other physical characteristics of the material can be found in Table 1.

**Table 1:** Characteristics of chrome steel beads.

Property	Value
Tensile strength [*MPa*]	2240.0
Yield strength [*MPa*]	2033.9
Modulus of elasticity [*MPa*]	203395.4
Density [*kg*/*m*^3^]	7833.4

In discrete approaches, it is important to make accurate measurements of the surface friction of the grains. Since the same chrome steel beads have been used previously [18, 19], the surface friction of the beads had already been measured using modified four-ball and sliding block tests. The resulting friction angles were 5.5° and 5.71°, with standard deviations of 1.26° and 2.22° respectively. However, the same sliding block tests were repeated on 4 mm diameter beads to validate the reported results: seven beads were glued together on a flat plane and placed exactly on top of another seven glued beads on a plane. The bottom plane was tilted slowly until the upper block of beads slid over the bottom one. The angle at which sliding began was measured. In addition, a load of 1 kg was applied on top of the top block of beads. By contrast with previous studies [19], the load on the top block reduced scatter in the results. A total of fifteen tests were performed (with loading) and an average angle of surface friction of 5.22° with a standard deviation of 0.71° was observed. This is close to the values reported by O’Sullivan [19]. In addition, the same tilting approach was used to measure the friction ratio for the boundaries. The same block of seven glued metal beads (with a 1-kg load on top) was placed on the bottom of the shear box and the shear box was slowly tilted until the block of metal beads began to slide. A total of thirteen tests were performed and an average friction angle of 5.88° with standard deviation of 1.14° was observed. Since a larger standard deviation was found for boundary friction and also because the value of the boundary friction was very close to that of bead-bead contacts, it was decided to use the bead-bead friction ratio for the boundaries as well.

The metal beads were packed in a rhombic configuration in nine rows in the direct shear box, resulting in 2163 metal beads in each sample. Particular attention was paid to sample preparation in order to create identical samples in terms of packing configuration. This stringent approach to sample preparation was necessary because:

- This configuration makes it possible to replicate exactly identical samples both in the simulations and in the laboratory and therefore to eliminate the uncertainties attributed to differences between the packing configurations of samples in the laboratory and in the simulations.
- From the statistical point of view, there is no need to prove the repeatability of the obtained results by extensive laboratory testing because the range of results will be very limited [35, 44–46]. This will be become clear in the Results and Discussion sections.
- The data from the numerical simulations will be compared with a narrower range in order to arrive at a more precise evaluation of the accuracy of Bullet.

**Fig 4:**
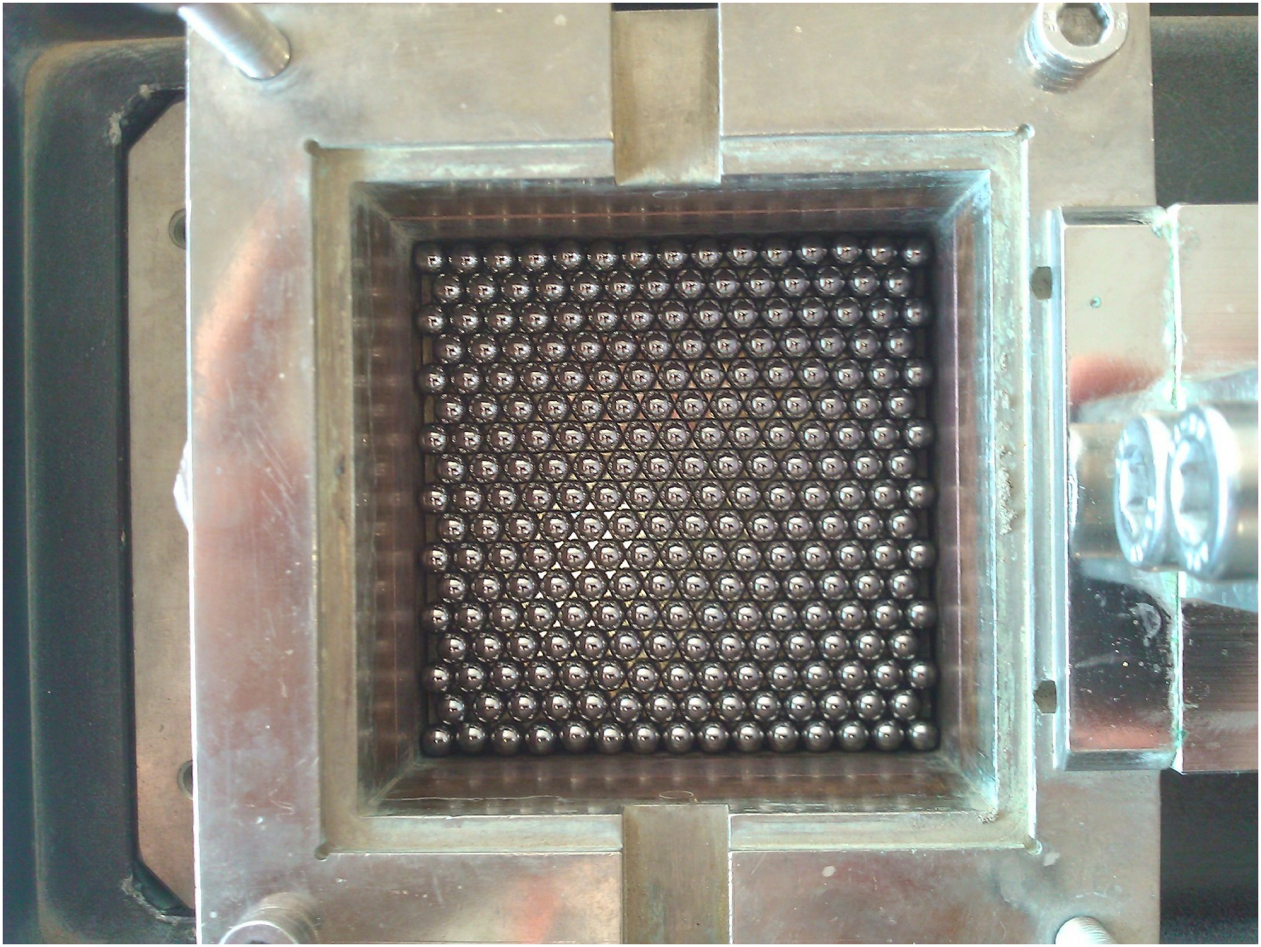
First row of metal beads placed in the direct shear box.

As stated above, the direct shear test was chosen for the testing method in this study due to its simplicity. The size of the direct shear box used in this study was 60×60 mm, and all the tests were performed at a constant strain rate of 6 mm/min. Fig 4 shows the first row of beads placed in the shear box.

## Simulations

The direct shear test code was written by the authors in C++ and it is freely available for interested researchers to test, use and develop [47]. The code was written for modular use. In other words, the user can change a wide variety of parameters such as direct shear box size, metal bead diameter, strain rate, surface friction of the beads, boundary friction, degrees of freedom (DOF) of different elements in the direct shear setup, etc. In addition, it provides a useful output stream for the data needed to draw stress-strain curves, chain forces, visualisations, etc. These features can be enabled/disabled in line with the user’s requirements.

It has been stated above that the input parameters for the contact law in a CD-based solver consist of the coefficient of restitution and the coefficient of friction. A value of zero was set for the coefficient of restitution since the coefficient of restitution does not affect the response of the system in dense granular systems where the dynamic regime does not prevail. Furthermore, experimental studies have shown that, in multi-contact systems, multiple shocks may occur and dissipate all of the kinetic energy present in very short time periods, and this can be simulated by setting this coefficient to zero [27, 48, 49]. A value of 5.22° was chosen for the coefficient of friction.

**Fig 5:**
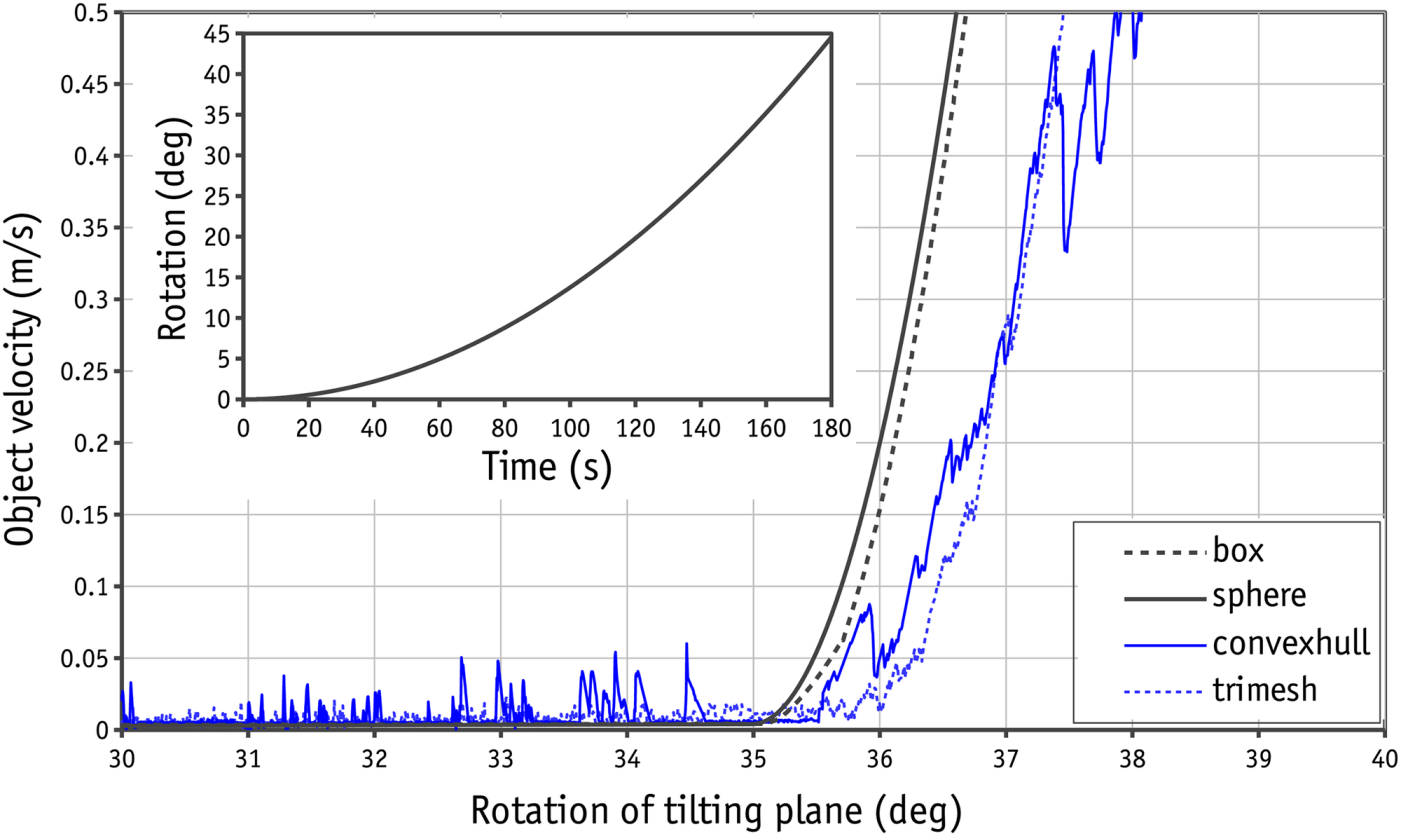
Velocity of different collision shapes in Bullet with regard to the rotation of tilting plane for interface friction angle of 35° (friction ratio of 0.7).

Energy is lost in two ways in the CD method: due to the elastic behaviour of objects in collisions, which is determined by the coefficient of restitution, and due to the movements/rotations of objects. In the direct shear test simulation, the regime is not highly collisional and so the second mechanism becomes important. Since the objects are assumed to be entirely rigid, the sort of movement/rotation of objects with respect to each other will be controlled mostly by the coefficient of surface friction. Prior checks were therefore needed to confirm the accuracy of Bullet for the calculation of friction forces in contacts. Several tilting plane tests were therefore conducted. It should be noted that Bullet takes the physical presence of the objects into account using collision shapes. The collision shapes available in Bullet are: sphere, box, plane, cone, cylinder, convex and trimesh. The last two were developed for arbitrary collision shapes using nodes and planes. Each collision shape has its own algorithm for contact resolution and so each should be evaluated individually. In the tilting plane test, three blocks made of box, convex and trimesh collision shapes were placed on a tilting plane. The dimension of the blocks was 2×2×0.5 units. A non-rotational sphere with a diameter of 2 units was also placed on the tilting plane to investigate the frictional behaviour of sphere collision shapes. The tilting plane was tilted slowly in a quadratic pattern with regard to time, as is shown in Fig 5, to prevent dynamic effects at the outset and during the test. The velocity of the objects was monitored to determine the angle at which the block began to slide. An example of the sliding response with a friction ratio of 0.7 for all the existing contacts can be found in Fig 5. As can be seen, the sliding threshold for the box shape and sphere was calculated correctly. In the case of convex and trimesh shapes, the sliding occurred at a slightly larger angle. Moreover, some fluctuations were observed with convex and trimesh collision shapes that can be attributed to the low iteration number of the solver (in Bullet, the default solver iteration number is 20 and the accuracy of the contact resolution is controlled by this parameter).

**Fig 6:**
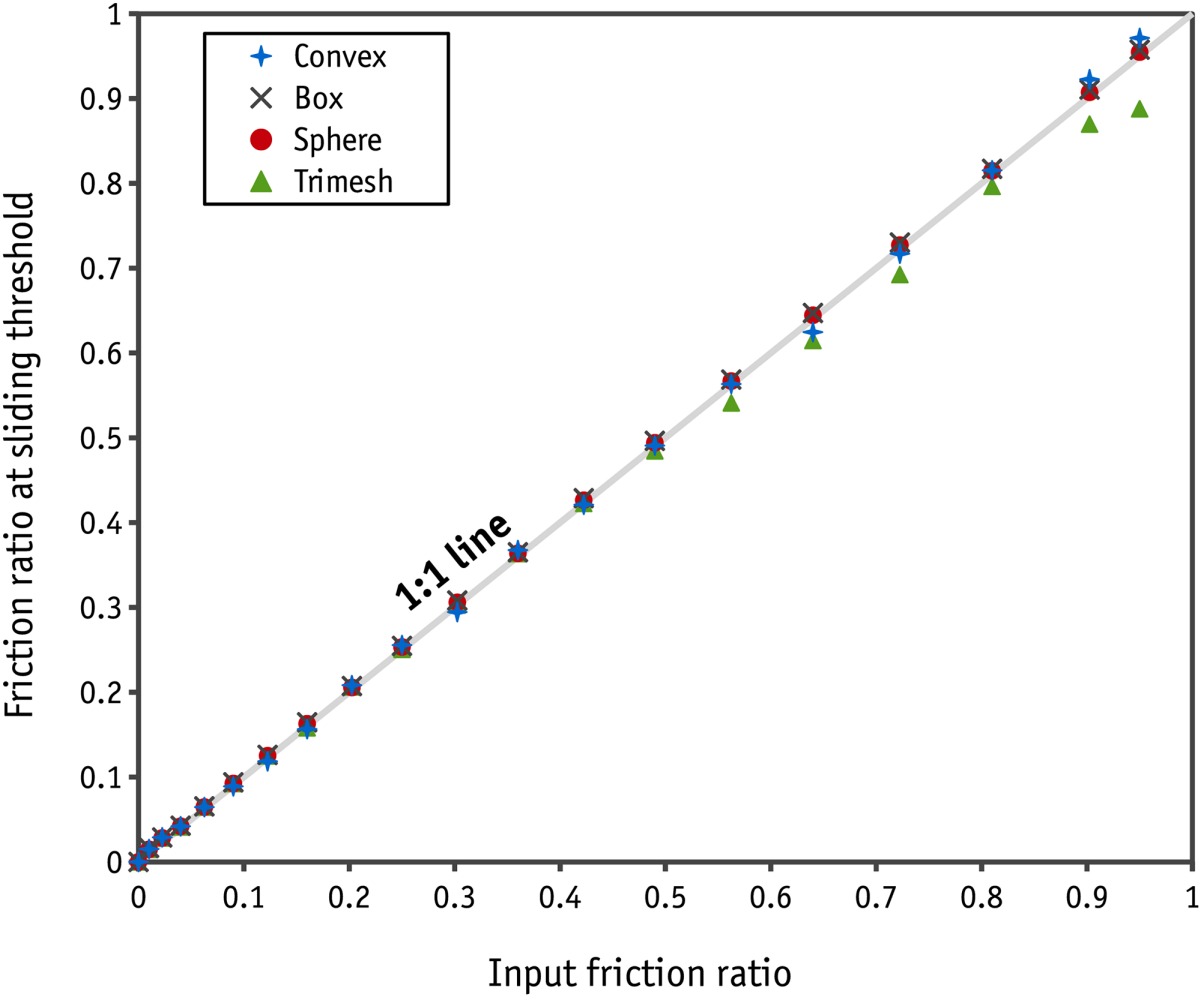
Input friction ratio vs the friction ratio at which slid occurs.

A similar test was repeated for different values of the friction ratio to determine the consistency of the results. The results are shown in Fig 6. Some inaccuracies were observed for a trimesh collision shape at large friction ratios. The results were consistent and accurate for the other collision shapes. From Fig 6 it can be concluded that using a trimesh collision shape makes the calculations less accurate, although the this discrepancy is not large. Indeed, increasing the iteration number in each step will improve the accuracy of the results.

**Fig 7:**
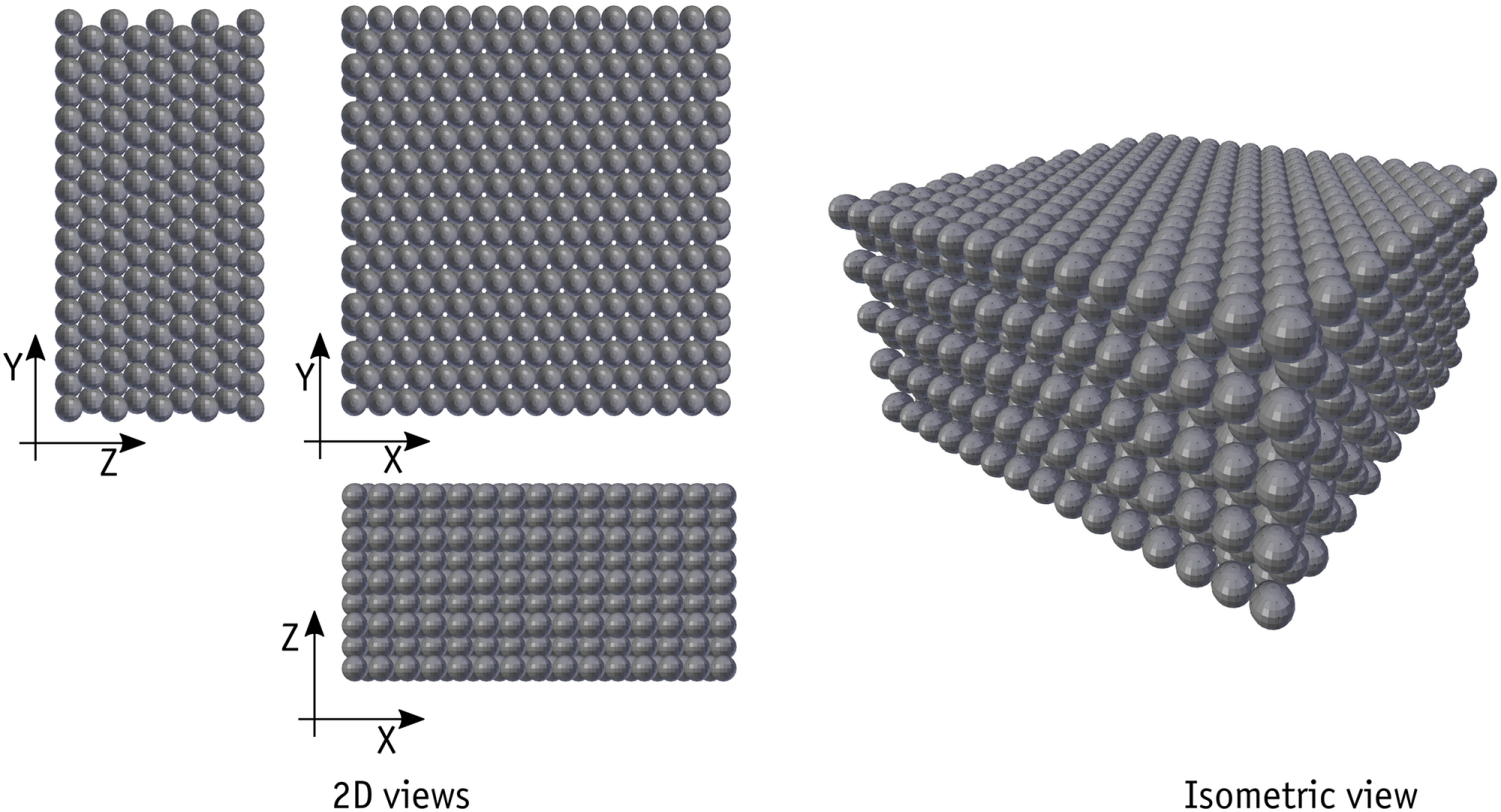
The modeled sample in Bullet.

To simulate the direct shear test on metal beads correctly, several steps were completed. Firstly, the basic global settings needed for Bullet solver to work were entered. The 4 mm metal beads were then generated using a sphere collision shape with the desired rhombic pattern in nine rows. The upper and lower shear boxes, along with the top plate, were subsequently modelled using box collision shapes. No DOF restraint was applied to the motion of the metal beads but DOF restrictions were set for the upper and lower boxes. The upper box rotations were restrained about all axes and movement in the vertical direction (here Z) was restrained as well. In effect, the lower box was modelled as a kinematic object, which means that it has a one-way interaction with other objects. In other words, its movement affects the movements of other objects but is not affected by other objects. When the sample was ready, the consolidation phase began by linearly applying a normal force in the centre of the top plate in order to reach the desired normal stress within one second. Particular care was taken to ensure that the sample was stable and reaches a state of equilibrium state at the end of consolidation. Immediately after the completion of the consolidation phase, the shearing phase began with the application of the prescribed movement of the lower box in the X direction at 6 mm/min for a total of 120 seconds. During the shearing phase, the normal load on the top plate was kept constant and the shearing force was monitored. The shearing force was measured in two different ways: the first approach consisted of modelling a very stiff spring that was attached to the upper box in the X direction and fixed to a static support at the other end. The shearing forces were measured on the basis of the changes in the length of the spring; in the second approach, the movement of the upper box was restrained in the X direction and the resultant forces exerted on the upper box were measured computationally. It was assumed that the friction ratio at the boundaries was the same as the friction ratio on the bead surface. To achieve greater realism, a very small rolling friction ratio of 0.0025 [50] was assigned to all the metal beads. The sample of beads simulated in 3D is shown in Fig 7. Note that two possible shearing patterns are possible given the configuration of the beads. A reasonable choice is to shear the sample along the weakest shearing direction (the X direction here).

**Fig 8:**
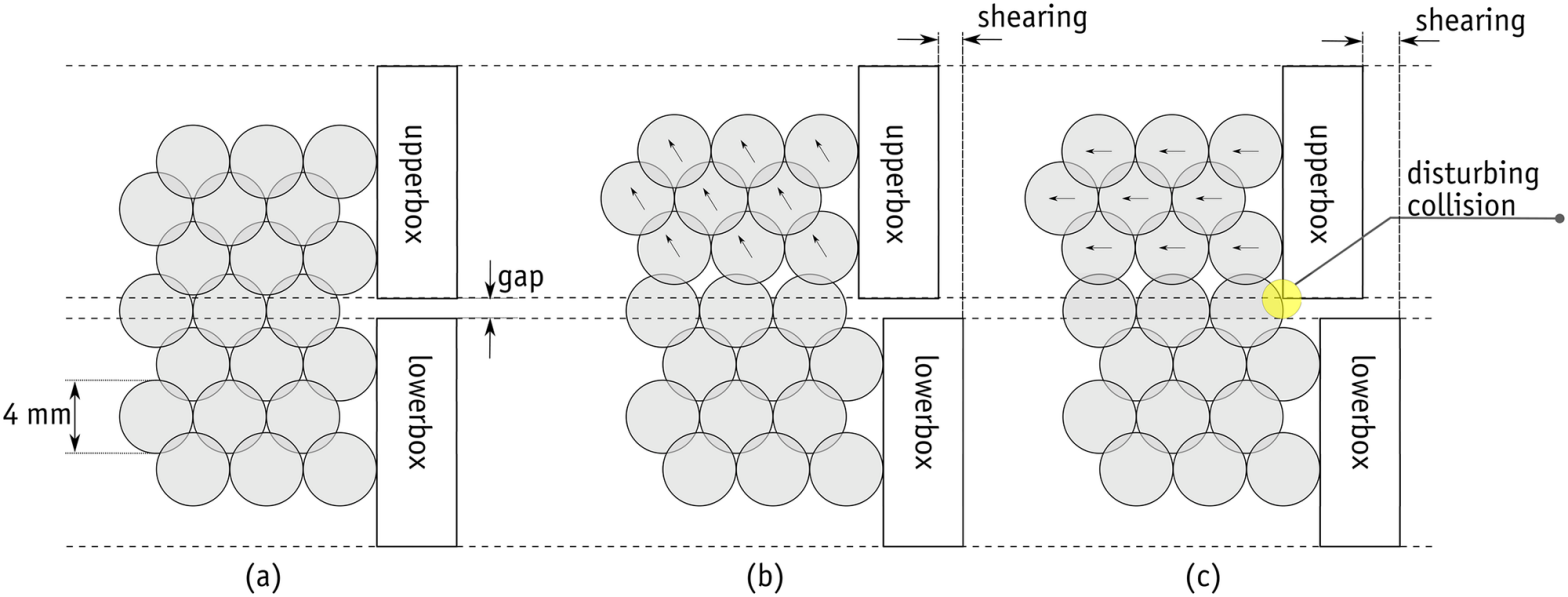
Magnified view of the edge of shear box: The effect of gap on the formation of failure plane.

Particular attention was paid to the magnitude of the gap between the lower and upper boxes and how it can affect the results. If the aim is to achieve straightforward bead-on-bead shearing, the gap should be large enough to prevent any interruption in the post-failure response. The amount of the gap is probably a trivial issue in direct shear tests on soil, especially when the ratio of the mean particle size to the shear box size is low. However, in the direct shear test with 4 mm beads, the edges of the upper box can interrupt the process of shearing and the formation of the shear band and failure plane. In the simulations, the gap was set at 3 mm to prevent any disturbance. It is important to note that there was no bead outflow in any of the tests. Fig 8 shows how the gap can affect and disturb failure plane formation on the edge of the shear box gap.

## Results and discussion

This section presents the results of the laboratory experiments, interprets them and then compares them with the results of numerical simulations using Bullet. In addition, the extra data provided by Bullet, which cannot be obtained from laboratory experiments, will be reviewed to understand the shear response in failure and post-failure states. The results of the simulations are then presented and discussed from the numerical viewpoint. Finally, a study of a direct shear test on real granular soil is presented to show that the critical state response can be captured using Bullet.

### Laboratory experiments

**Fig 9:**
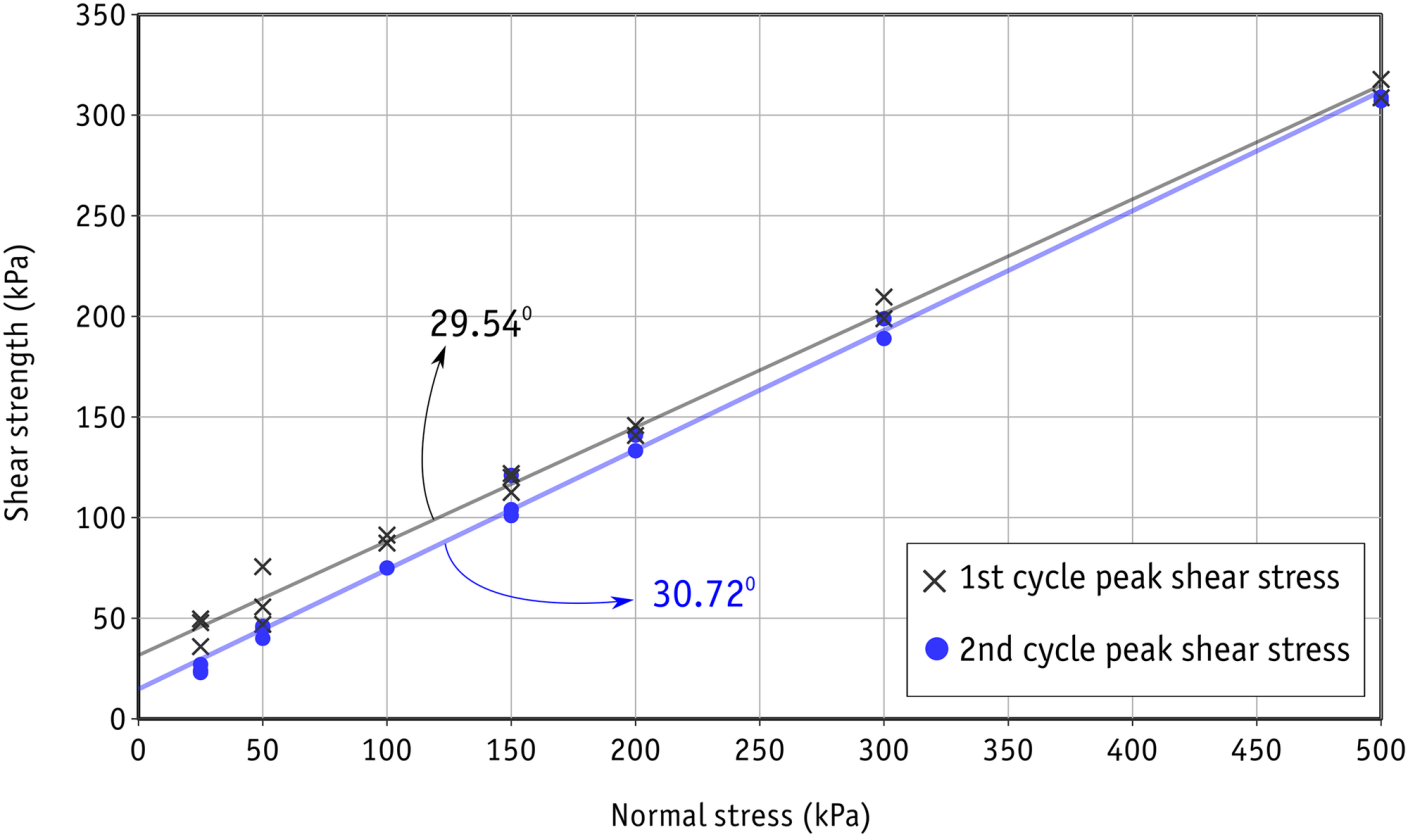
Shear strength envelope from the laboratory experiments.

**Fig 10:**
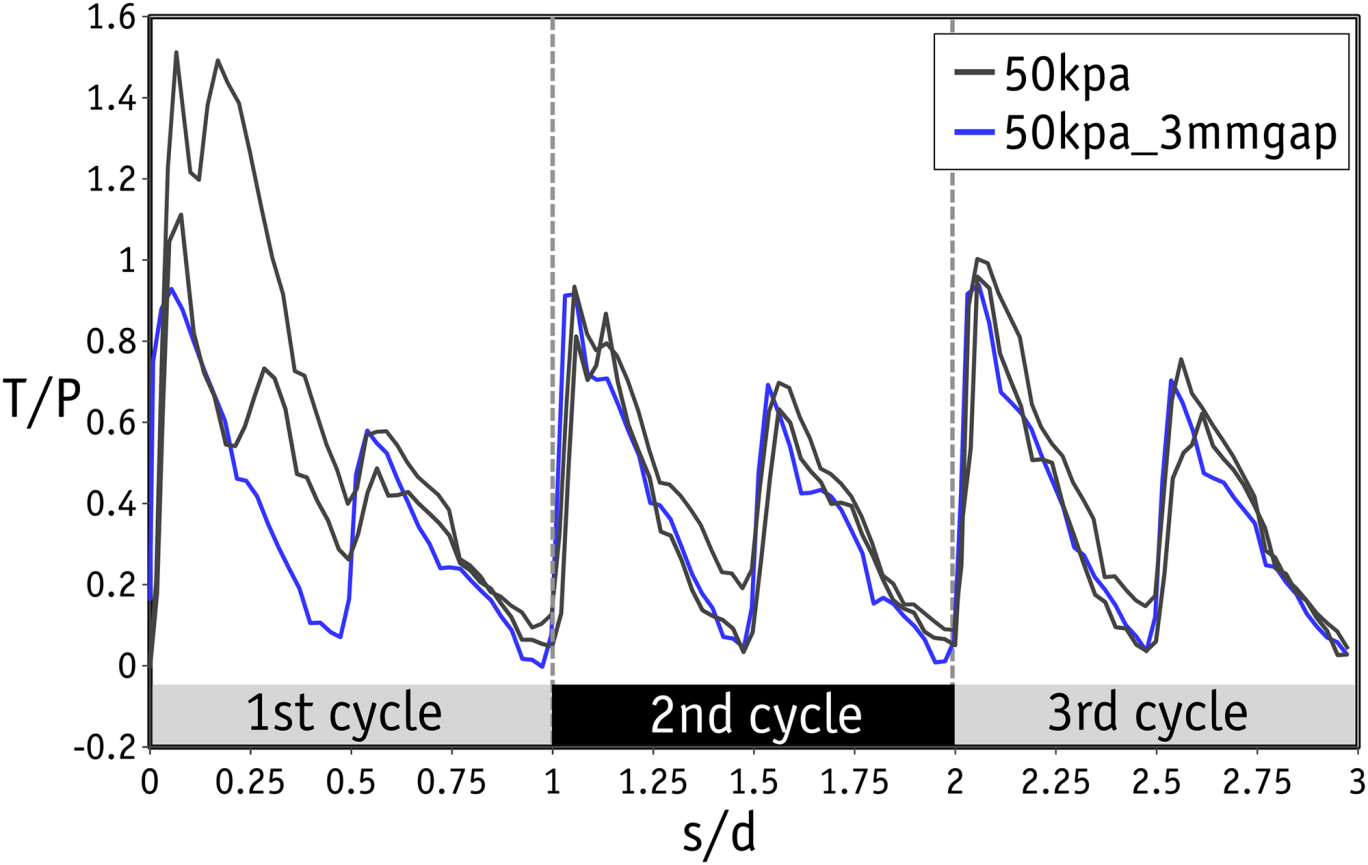
Stress-strain curve for the laboratory tests under normal stress of 50 kPa.

A series of direct shear tests were conducted on the uniform-sized metal beads packed in a rhombic configuration under normal stress levels of 25, 50, 100, 150, 200, 300 and 500 kPa. A wide range of stress levels were selected to establish a proper picture of how the shear response changes with stress level. Eighteen tests were performed in total. The first peak in shear stress observed in the stress-strain graph for each test was considered to represent the ultimate strength of each sample. Accordingly, the familiar shear strength envelope can be drawn as shown in Fig 9. As can be seen here, the data points are almost aligned in a linear trend, which results in an average friction angle of 29.54° by linear regression. Nevertheless, a relatively wider scatter is present in the data at lower stress levels. This scatter in results at low stress levels in direct shear tests has been reported previously in the literature [51, 52]. With regard to the amount of total shear displacement (12 mm), which is three times the diameter of the metal beads, three similar cycles of repeated shear response were expected in the stress-strain curve of each test. This scatter was found only at the peak shear stress in the first cycle, and the range became limited in subsequent cycles. The stress-strain curves for the tests performed under 50 kPa are shown in Fig 10, where *s* is shear displacement and *d* the diameter of metal beads and *T* and *P* are the shear and normal stresses respectively. *T*/*P* can also be seen as the mobilized coefficient of friction in the sample since there is no cohesion between the metal beads. Fig 10 shows the results of two tests in identical conditions (black lines) along with a test (blue line) with a controlled gap between the upper and lower boxes of 3 mm.It should be noted that the cohesion represented by the intercepts of the shear envelopes shown in Fig 9 is due to the interlocking of the beads in the rhombic configuration.

The second shear strength envelope can therefore be drawn for the consistent values of peak shear strengths obtained in the second cycle. Figs 9 and 10 show clearly that scatter is also limited at low stress levels. An average friction angle of 30.72° was obtained with linear regression for the second cycle shear envelope.

**Fig 11:**
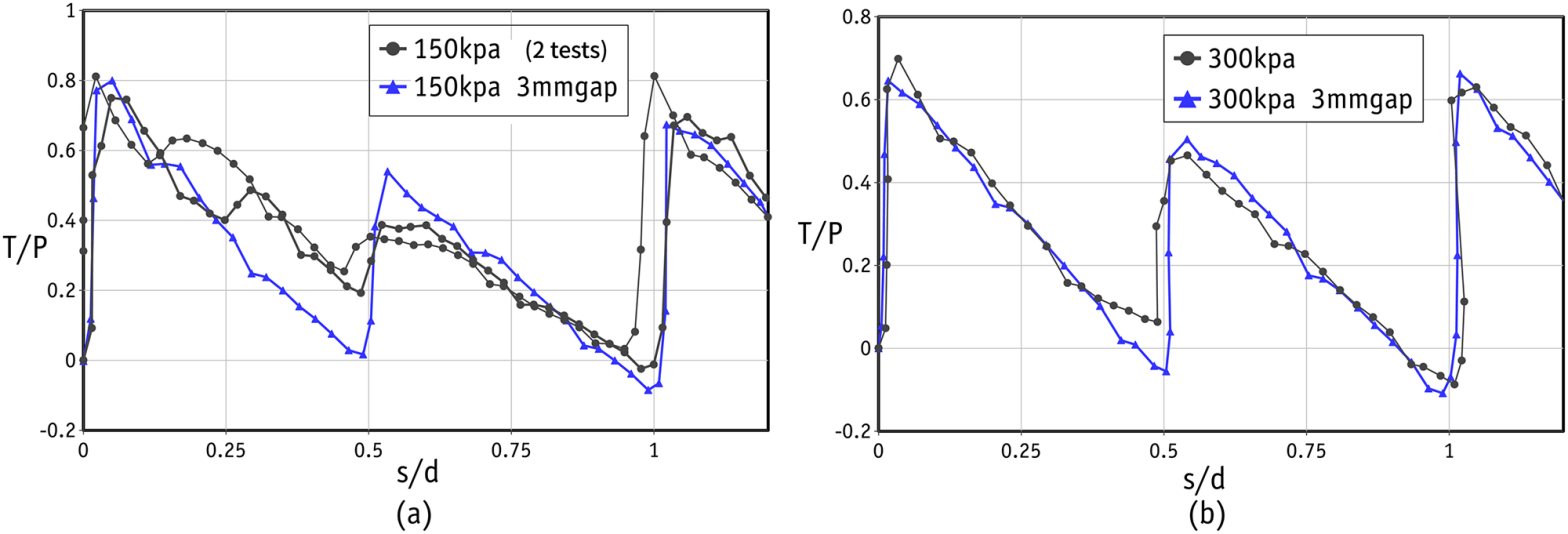
Stress-strain curve for the laboratory tests under normal stress of 150 and 300 kPa.

**Fig 12:**
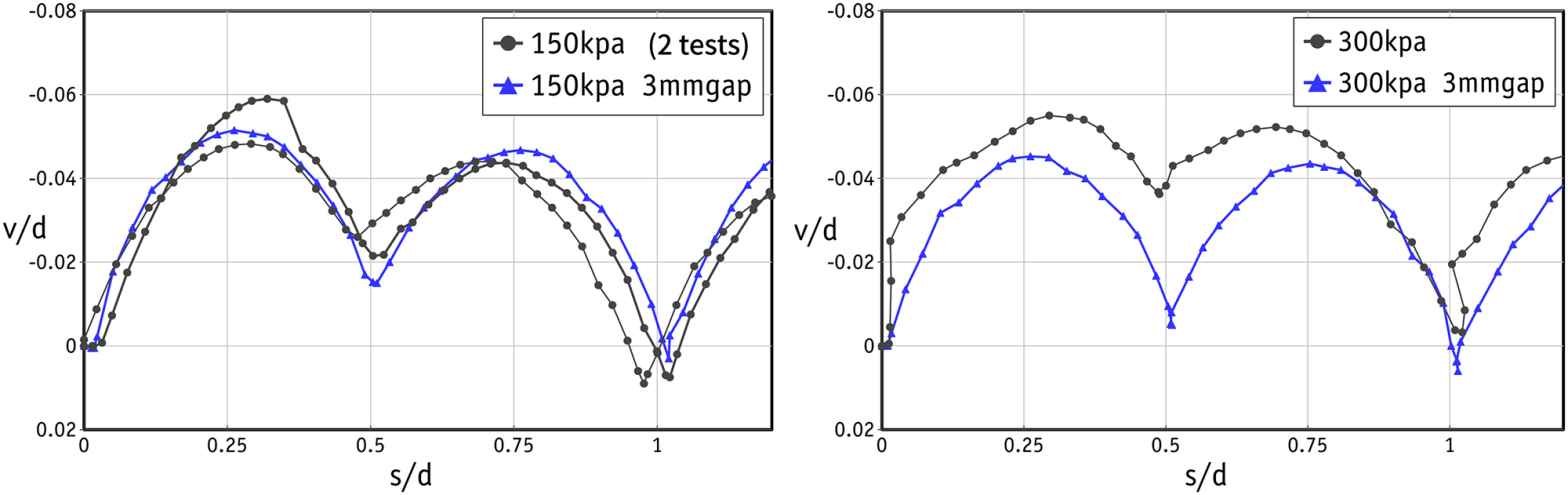
Dilative response of the samples for the laboratory tests under normal stress of 150 and 300 kPa.

Apart from the scatter in the tests under low normal stress levels, the shear response in the rest of the tests was found to be very similar in all three cycles. In the remainder of this study, therefore, we will look at the shear response in the first cycle. The shear responses in three tests with normal stresses of 150 kPa (2 tests) and 300 kPa (1 test) are shown in Fig 11. The shear response was very similar in each repeated test, confirming that the results are reproducible. This can be attributed to the highly controlled sample preparation using precision beads. The peak values occurred at very small strains and were followed by a sharp fall in shear stress, indicating that the sample failed in a brittle way. The dilative response of the sample at the same stress levels is shown in Fig 12. Once again, there are two peaks in all cases at the strains of 0.25 and 0.75 in each cycle. The amount of dilation measured in all tests was comparable and varied between 4 and 6%.

**Fig 13:**
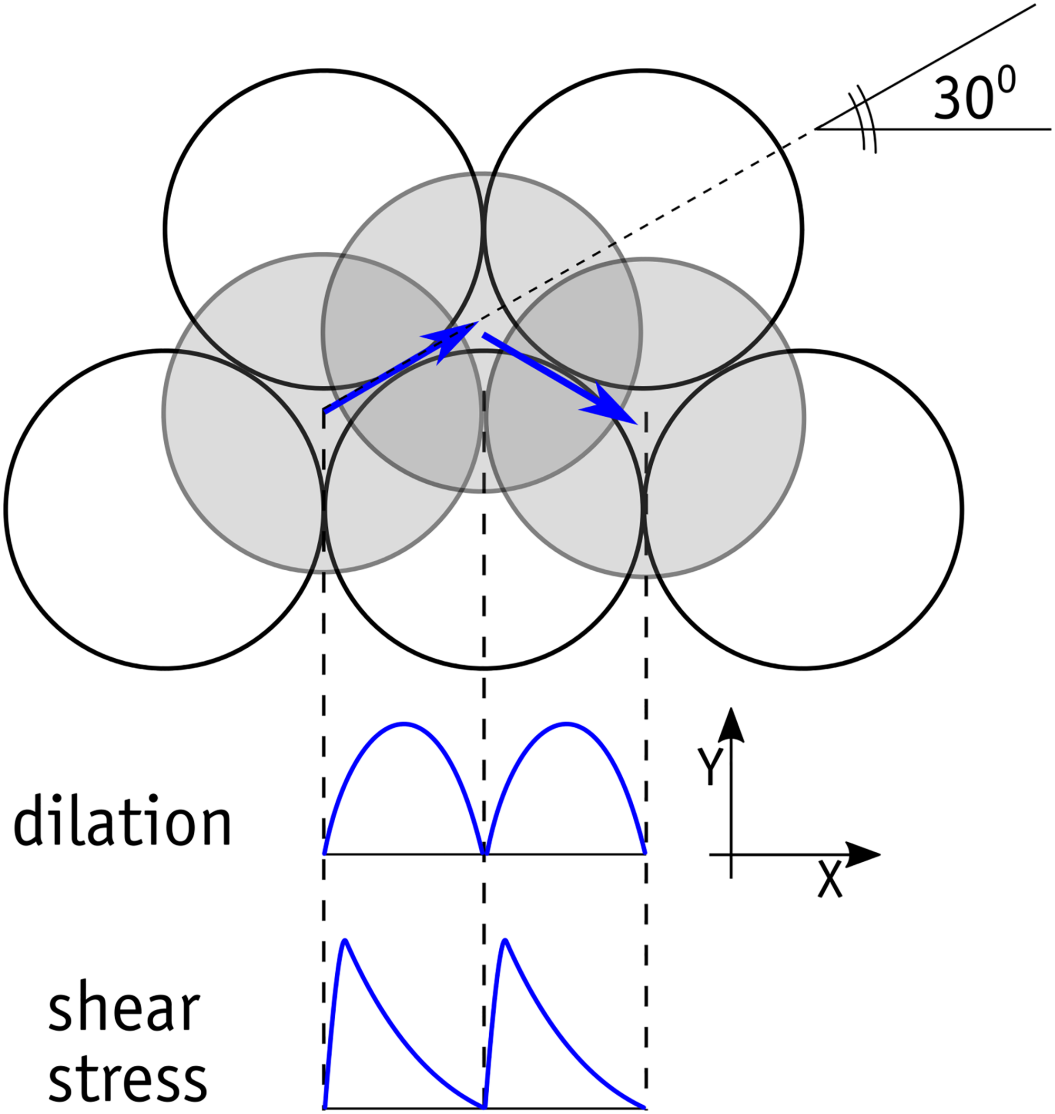
Movement of a bead subjected to horizontal and normal load.

**Fig 14:**
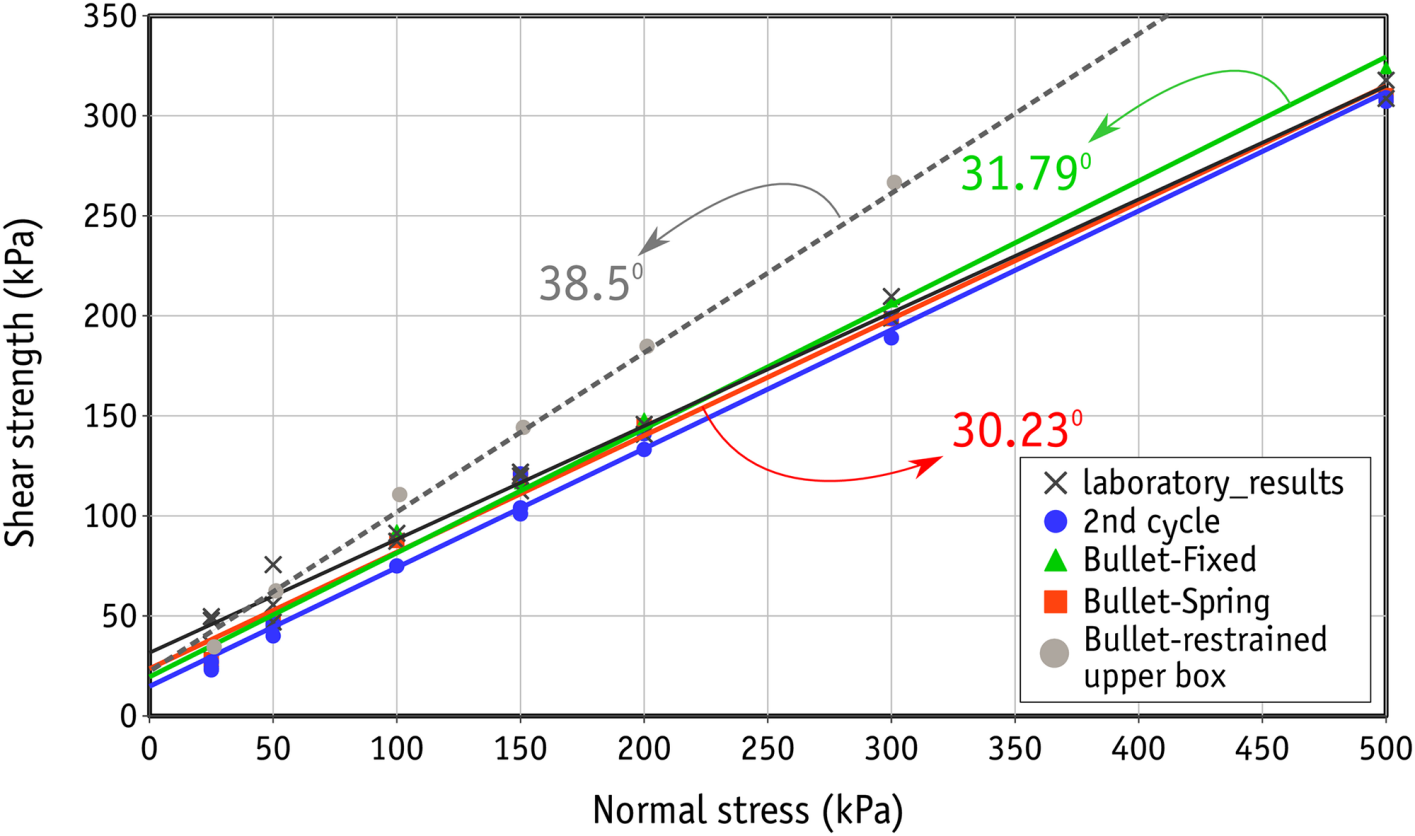
Comparison of shear envelopes obtained from numerical results and laboratory experiments.

In Figs 11 and 12, there are two peaks in the shear stress and dilation curves in each cycle. In Fig 12, *v* represents the vertical downward displacement of the sample. Given the configuration of the metal beads in the direct shear boxes, such behaviour implies that shearing was not entirely in the X direction. In each row of beads in the rhombic configuration, there are troughs and bumps due to the spherical shape of the beads. Releasing the translational DOF in the upper box in the direction perpendicular to the shearing directs the beads in the upper row onto the notches between the beads below. On a larger scale, the upper box will move in a zig-zag in the horizontal plane and shear in the weakest failure direction, as is shown Fig 13. Obviously, if the movement of the upper box in the Y direction is restrained, a larger shear stress and a larger mobilized friction angle are to be expected, as shown in Fig 14.

The laboratory experiments were conducted in two separate phases. In the first phase, the size of the gap was not controlled and the test followed the standard procedure. In the standard procedure, after the application of the normal force, two screws were used to make a small gap in order to prevent the results being affected by unwanted friction between the upper and lower boxes. The gap in the standard procedure was roughly 1.5 mm. With such a small gap, the edge of the upper box can touch the metal beads and distort the results (as explained in Fig 8). It is important to note that this distortion is found in the post-peak response, and the number of peak stresses will be independent of the size of the gap. It was therefore decided to repeat the tests with a controlled gap of 3 mm to ensure that the edge of the upper box would not touch the metal beads in the lower row of the failure plane. The results of these tests are shown by the blue line/data points in Figs 10, 11 and 12. The data for the gap-controlled tests showed that, as expected, there were no changes in the peak shear stress values. However, the level of shear stress in the mid-cycles (*s*/*d* = 0.5, 1.5 and 2.5) was reduced to zero or even negative values. This can be seen as an indication that unwanted friction due to the contact between the edge of the upper box and the metal beads had been prevented. In addition, the additional dilation of the sample resulting from this was eliminated. It is important to note that the test with a controlled gap did not affect the scatter in the first cycle peaks in the tests with low normal stresses.

It is said that the direct shear test is popular due to its simplicity for the purposes of geotechnical engineering. However, the results of laboratory tests on metal beads have revealed that, in the case of tests for validation purposes, particularly with perfectly-shaped and perfectly-sized granular material, extra attention needs to be paid to delicate matters such as the size of the gap and the DOF of the upper box. In addition, previous experimental studies with direct shear tests showed that many factors can affect the results of the tests, such as the sample size, gap, the boundary friction between shear box and granular media, and the DOFs of the elements in the direct shear setup [46, 52–54].

### Simulation results

In this section the results of the numerical simulations using Bullet will be compared with those of the laboratory experiments. The shear strength envelopes that derived from the numerical simulations are shown in and compared with the laboratory results in Fig 14. Unlike the laboratory results in which some scattering in the peak shear strength in the first cycles were observed, no scattering was monitored in the simulation results. As a consequence, for each case of simulation (derivation of shear force using a reactive spring, denoted as *Bullet-spring*, and by calculation of resultant horizontal forces acting on the upper box, denoted as *Bullet-fixed*) one shear envelope is drawn. Both shear envelopes are in a good agreement with those of laboratory experiments, resulting to average angle of friction of 30.23° and 31.79°, for Spring and Fixed cases, respectively. However, for the fixed case, at large stresses the calculated peak shear strength was larger than the laboratory results. In addition, an average friction angle of 38.5° was observed for the case if the translational movement of the upper box is restrained in the direction perpendicular to the shearing.

**Fig 15:**
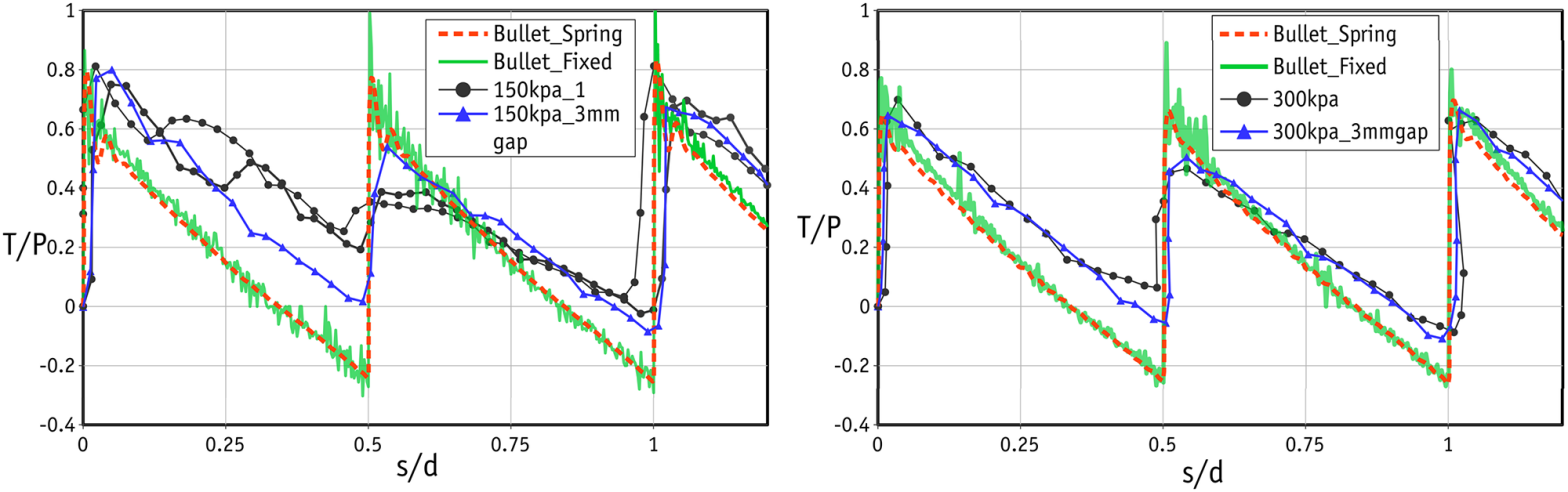
Comparison of stress-strain curves for numerical and laboratory results.

The stress-strain curves for normal stresses of 150 and 300 kPa are shown in Fig 15. The curves for the two simulation cases match well but the main difference is that, when the forces are derived computationally (in the Bullet Fixed case), more fluctuations are found when deriving the shear force from spring. The fluctuation can be attributed to the nature of the CD approach, in which the solver uses larger time intervals. As noted before, no scatter was found in the simulation results and so the scatter in the laboratory results could be a completely experimental issue. A possible reason could be the friction force mobilized between the internal planes of the upper shear box and the metal beads during the generation of the gap. Since this scatter is found only at low stress levels, even small levels of mobilized friction could alter the global response. As can be seen in Fig 15, increases and decreases in shear stress match those in the laboratory data. Nevertheless, Bullet did not fully capture the post-peak response. In the post-peak stage, the shear stress derived by Bullet was lower than in experiments. Although repeating the laboratory tests with a gap of 3 mm improved the match between numerical and laboratory results, the difference in the post-peak shear stresses was still not negligible. Previous attempts by O’Sullivan et al. [18, 45] have also had little success in terms of capturing the global post-failure response. They argued that this discrepancy was attributable to a variation in the real coefficient of friction between particles and boundaries. However, even in further simulations with variations in boundary friction, it was not possible to capture the post-peak response properly. The authors of the present paper believe that this discrepancy is a result of the damped resiliency of the load cell used in the direct shear setup. Reproducing exactly the same properties in the simulations is very challenging, particularly in the current case, in which a large stress was mobilized prior to the instant peak shear stress, followed by a significant reduction. A proper post-peak response can probably be captured when the ultimate shear stress occurs in a much wider range of strains (in other words, when there is ductile failure) [45].

**Fig 16:**
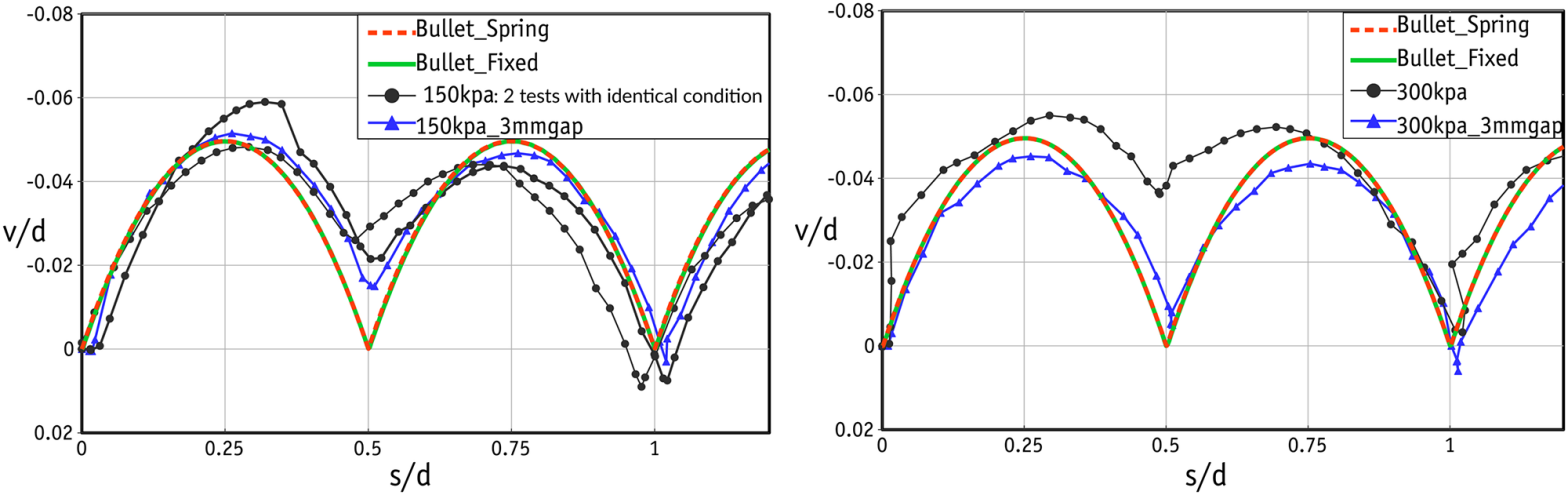
Comparison of dilation curves for numerical and laboratory results.

The dilation responses of the samples subjected to the normal stresses of 150 and 300 kPa are shown in Fig 16. Clearly, both simulations led to very similar values. The simulation data closely match those from the laboratory experiments at both normal stresses. It can be deduced that the dilative behaviour is independent of the stress level since the metal beads are rigid enough and are therefore not deformed easily by high loads. As with stress-strain curves, the laboratory experiments repeated with a controlled gap of 3 mm produced a better match with numerical data.

**Fig 17:**
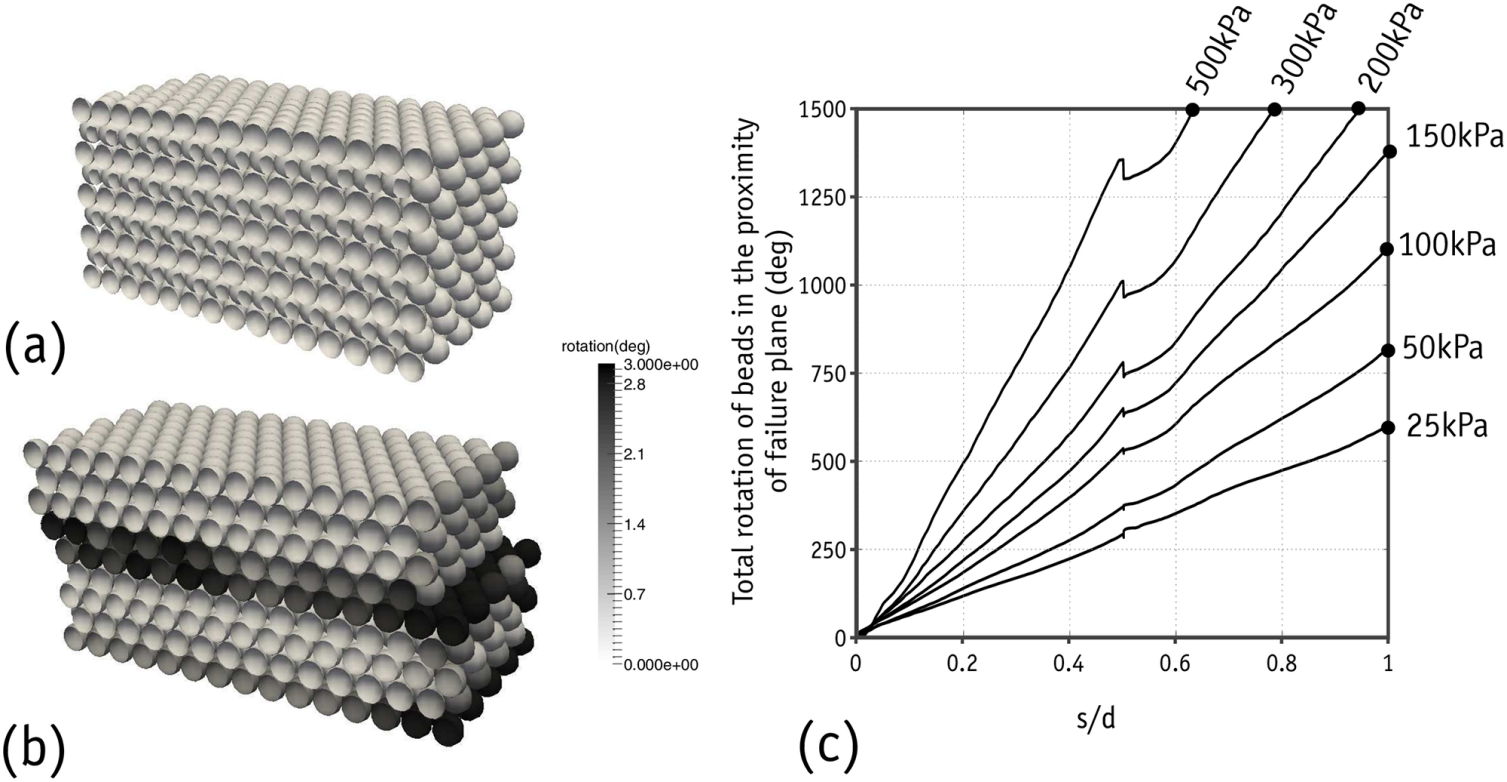
Rotation of metal beads: Prior to shearing(a); at shearing of s/d = 1(b); total rotation of beads located in the proximity of failure plane(c).

**Fig 18:**
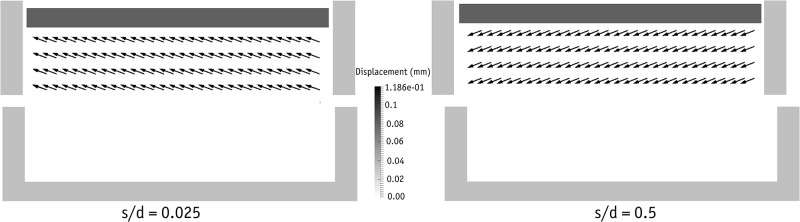
Displacement vectors of metal beads during shearing for the sample under 150 kPa.

In addition to the macro-scale results discussed above, Bullet can provide further details at the particle scale about areas such as object movement, rotation, force, etc. This detailed information can, if presented well, provide the user with a better understanding of the mechanisms prevailing in the sample during shearing. The cross-section of the sample subjected to 150 kPa normal stress is shown in Fig 17(a,b), where the beads are shaded to show the magnitude of rotation prior to shearing and at the end of the first cycle of shearing. As can be seen, the beads located in the fifth and sixth rows have rotated. The rotation of the beads shown in Fig 17 and the displacement vectors of the beads shown in Fig 18 clearly show that the sample was divided into two blocks of beads and sheared along the interface between the fifth and sixth rows. Since the magnitudes of the displacement vectors were almost the same, there was no particle rearrangement in the upper and lower blocks. This failure mechanism was found to be independent of the stress level and a similar trend was observed at different stress levels. However, the level of rotation was found to be dependent on the stress level, as shown in Fig 17(c). Since bead rotation near the failure plane was of interest, the total rotation levels were calculated for the fifth and sixth rows.

**Fig 19:**
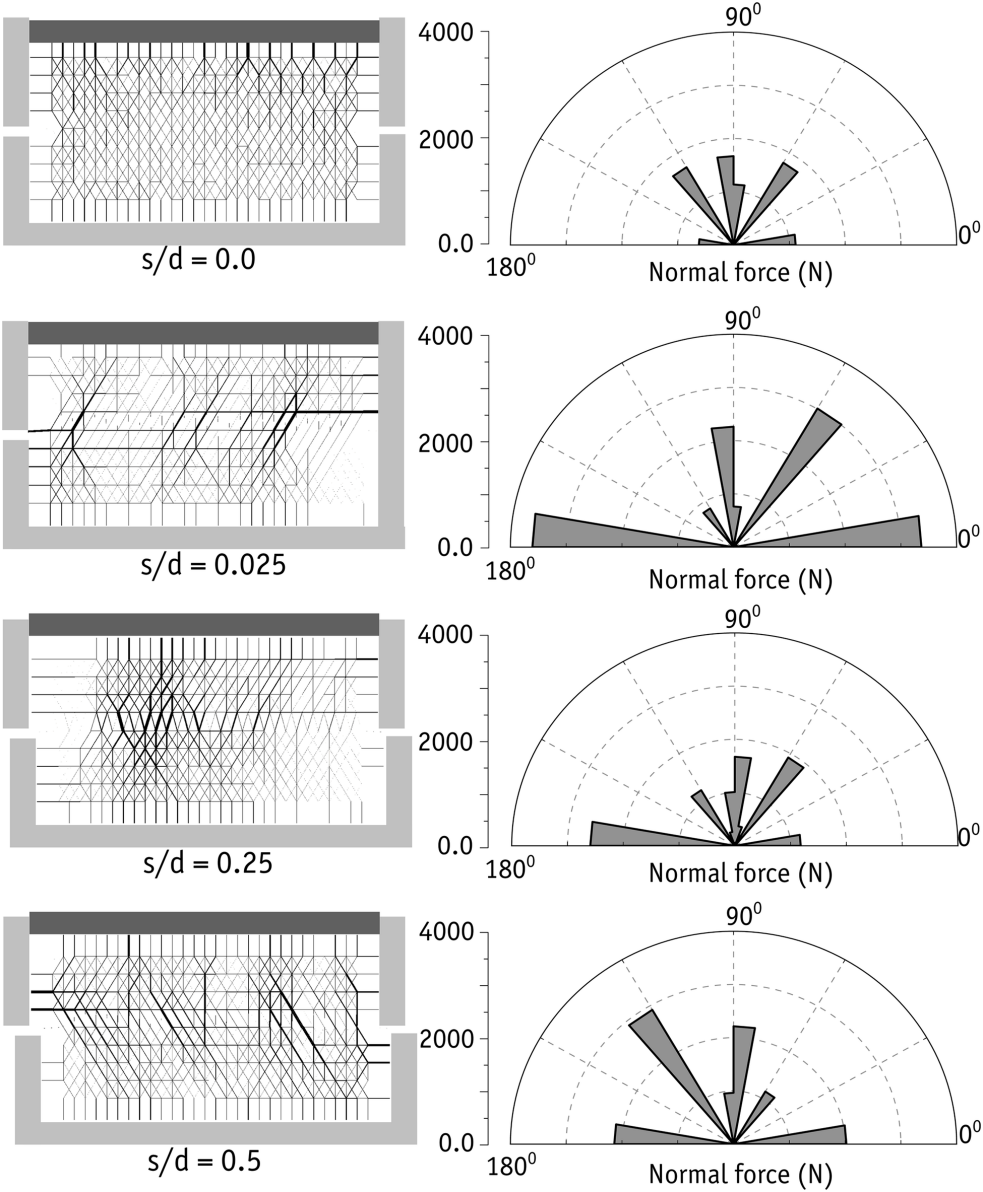
2D projected view of the normal chain forces at different stages of shearing (left) and the corresponding accumulated normal forces.

Fig 19 shows the network of normal forces in different stages of shearing, along with the corresponding distributions of polar contact forces. The forces are distributed evenly prior to shearing throughout the domain. As soon as shearing begins, major horizontal chain forces form to resist the shearing. These forces transfer the shear forces through the beads located on the failure plane and so the major chain forces diverge diagonally in the failure plane. Depending on the magnitude of shearing displacement, the direction of the major chain forces in the failure plane changes. As seen above in Fig 13, the metal beads located on the upper side of the failure plane undergo two successive periods of rolling-up and falling-down in each cycle of shearing. When the shear displacement is large enough to place the metal beads above the failure plane in a falling-down position, the chain forces in the direction opposite to shearing become stronger, producing a negative shear stress value. These negative shear stresses were observed in the laboratory experiments but the numerical data contained higher negative values.

In all the simulations presented above, a time increment of 0.01 seconds and 70 iterations per step were adopted. Each simulation case lasted approximately 20 minutes for 121 seconds (1 second of consolidation and 120 seconds of shearing) of real simulation time on a system with 32 GB of RAM, Intel Corei7-3820 3.6 GHz processor (single core was used). Given the results presented here and the considerations given in the discussion, Bullet can be used as a discrete simulation tool for the modelling of granular systems.

### Application to a real granular soil

A uniformly graded sand was selected for the simulation of the direct shear test on real-like soil. The largest grain size, *D*_*max*_, and the smallest grain size, *D*_*min*_ in the generated sand were 4.0 and 1.8 mm respectively. The surface friction ratio of the modelled sand grains was assumed to be 0.7 and the friction between the sand on the one hand and the shear box and the top plate on the other were assumed to be the half of the grain surface friction.

In order to model the geometry of the sand grains appropriately, a cuboid of soil with the same volume as the sand supposed to be included in the shear box was subjected to Voronoi discretisation to generate irregularly-shaped polyhedral objects. However, the size range, the degree of uniformity of the grains, the degree of angularity (defined by the geometrical scale factors of each grain in the x,y and z directions) and the proportion of angular soil in the total soil mass were fully controlled. The discretized objects generated were very similar to the real ones, as has been reported in a previous study by the present authors [35]. A total of 3674 polyhedrons were generated and a unique convex collision shape was assigned to each individual grain.

To make the samples in the simulations, the discretized objects were mixed thoroughly and poured into a 60x60 mm shear box. The samples were prepared in two different densities. To make the loose sample, the grains were dumped into the shear box and the grains on top were evenly distributed to make an almost flat surface to receive the top plate. By contrast, during the production of the dense sample, the surface friction between the objects was temporarily reduced before the grains were poured into the shear box to obtain a denser configuration at the end. After the grains were settled in the shear box, the surface friction was set to the original value (i.e. 0.7). Both samples were subjected to a normal stress of 150 kPa in the consolidation phase. The void ratios of the dense and loose samples at the end of the consolidation phase were 0.41 and 0.61 respectively. However, the density obtained for the dense sand was not the highest density which can be achieved numerically, the aim being to demonstrate that Bullet can capture the critical-state-type response of the soil at this void ratio. The movement of the upper box was restrained in the horizontal direction perpendicular to the shearing direction, and the shearing was induced by moving the lower box at a rate of 0.6 mm/s for 20 seconds. The time increment adopted for these simulations was 0.001 second and the number of solver iterations per step was set to 70. The dense and loose samples were made with a total number of 3673 and 3142 polyhedrons, respectively. The simulation time was 20 seconds as stated previously, however, it took 20 minutes of CPU time using a single core of Intel Corei7-3820 3.6 GHz processor.

**Fig 20:**
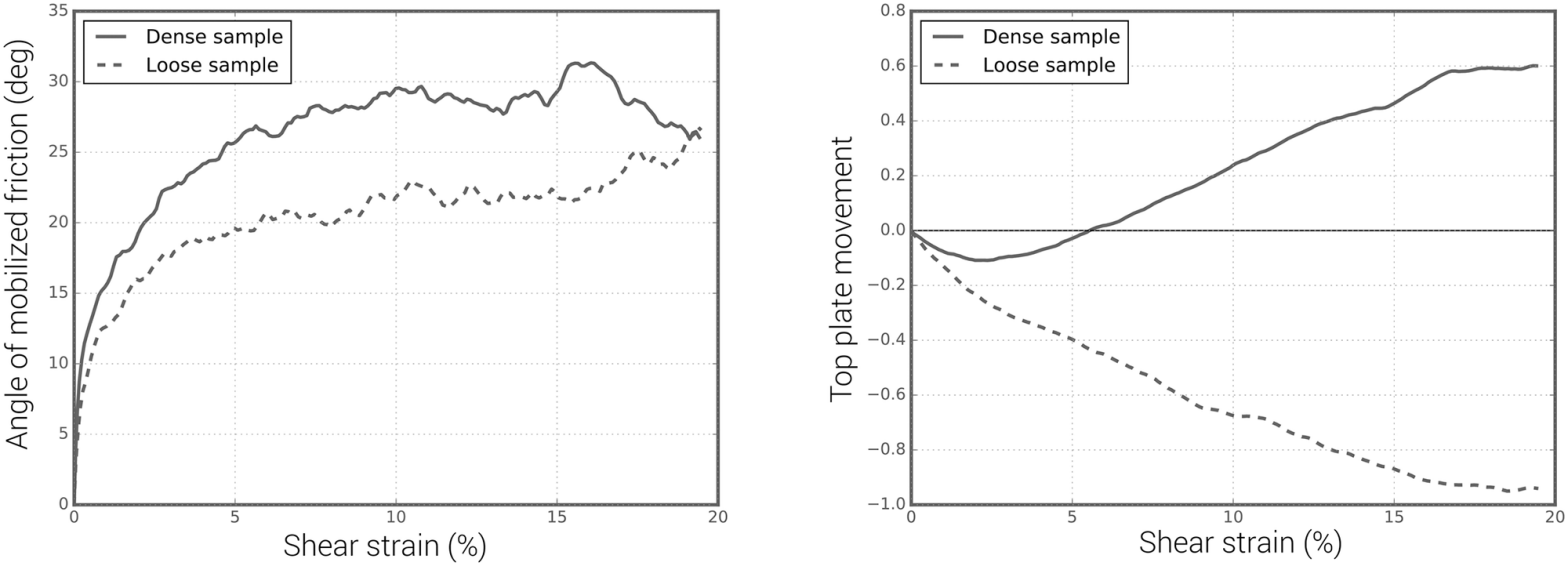
Angle of mobilized friction and dilation curves for dense and loose samples.

The angle of mobilized friction and the movement of the top plate are shown in Fig 20, where it can be seen that the dense sample exhibited higher initial shear stiffness and a higher peak angle of mobilized friction than the loose sample. The mobilized friction angle in both samples tended to converge to 26.5° at strains exceeding 15%. As was expected, overall dilative behaviour was seen in the dense sample, which tended to contract initially at shear strains of up to 2.5% shear strain, and to dilate continuously at larger shear strains of up to 16.5%, after which the volume of the sample remained constant. By contrast, the loose sample tended to shrink progressively at shear strains up to 16.5% and the volume did not change at larger shear strains.

**Fig 21:**
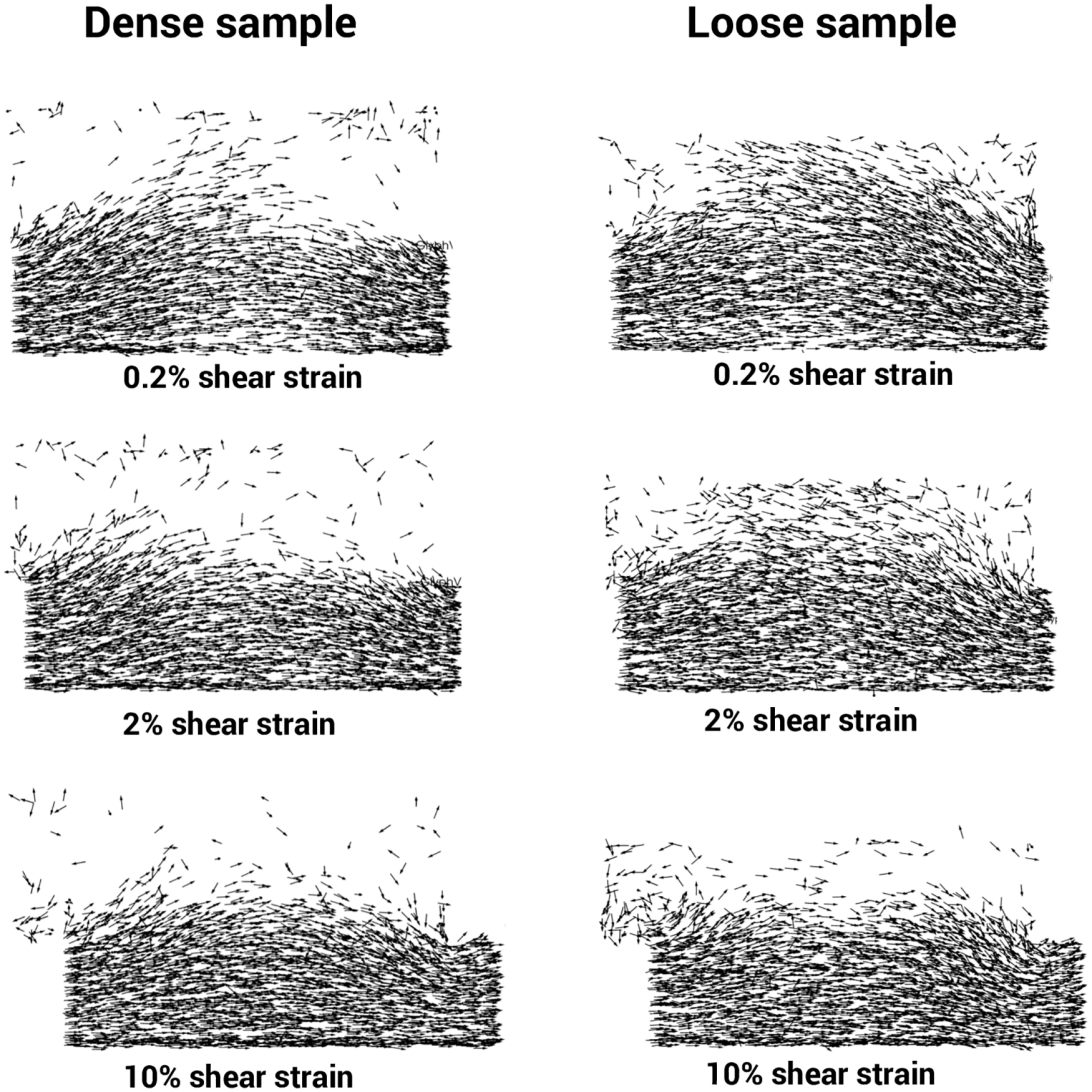
Displacement vector field of the sand grains for dense and loose samples (only the vectors larger than 0.06 mm are visualized).

Fig 21 shows the displacement vectors of the dense and loose samples at 0.2%, 2% and 10% of shear strains. It should be noted that only displacement vectors larger than 0.06 mm are visualized. Given that the lower box was subjected to the prescribed shearing displacement, there was a smaller level of displacement of 0.06 mm in the majority of the sand grains located in the upper box of the sample. Nevertheless, a significant number of sand grains in the loose sample in the upper box were subjected to a displacement larger than 0.06 mm. This was not the cases in the dense sample, which indicates that a large passive block was formed in that sample that generated additional resistance to shearing at the top-right side of the shear box. When subjected to larger shear strains (10%, for example), the loose sample contracted more and there was less grain movement in the upper box.

**Fig 22:**
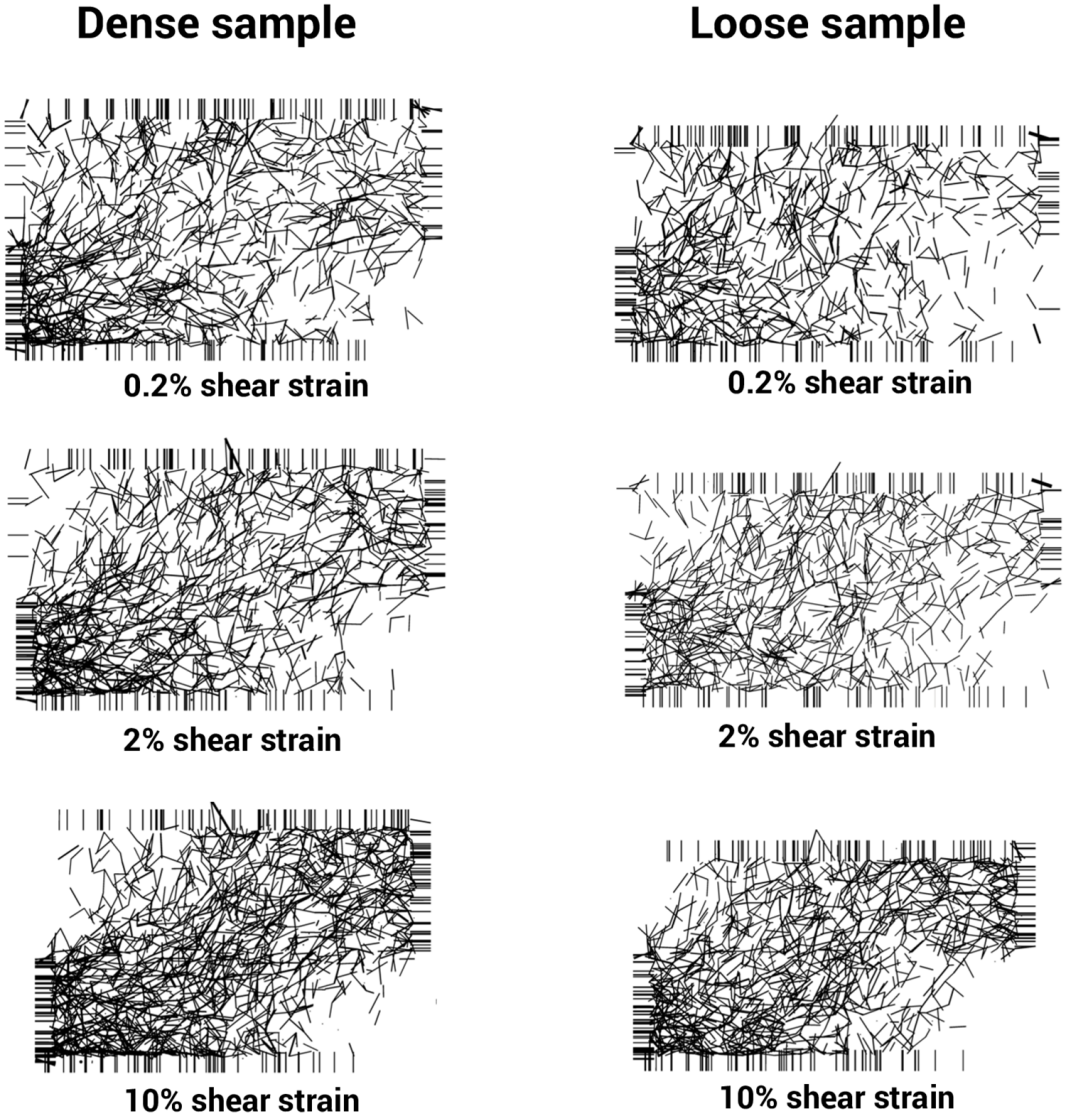
Chain force network of the sand grains for dense (left) and loose (right) samples (only the forces larger than 1.7 N are visualized).

The chain force networks in the dense and loose samples are shown at different stress levels of 0.2%, 2% and 10% in Fig 22. It should be noted that only the forces larger than 1.7 N are visualized in Fig 22. In addition, the line thicknesses are scaled by the amount of normal force calculated between two individual grains. A denser bunch of chain forces was seen in the whole of the dense sample. However, chain forces were densest in the bottom-left corner of the dense samples. This was also the case at different shear strains. Overall, apart from the density of the chain forces, there was no very clear difference between the chain force networks in the dense and loose samples.

## Concluding remarks

This paper aimed to demonstrate that the Bullet physics engine can be used to simulate granular systems. Physics engines were first developed for use in the animation and gaming industries but their unique features led to their use by many researchers in various fields. Depending on the type of physics engine, they may be capable of handling rigid body systems, soft bodies and fluids in a fast, stable and efficient manner. The accuracy of Bullet was investigated in the present paper for the purposes of rigid body dynamics: a series of validation tests were carried out on uniformly-sized metal beads in direct shear tests. The metal beads were tightly controlled in terms of diameter and sphericity, and packed in a rhombic configuration in a shear box measuring 60x60 mm before being subjected to shearing under various normal stresses. The same procedure was followed in Bullet as well. The comparison of the laboratory results with those derived from simulations demonstrated that Bullet captured the peak strengths very well. However the exact post-failure response was found to be difficult to capture. In addition, we investigated the mechanism of shearing on the basis of the detailed information provided by Bullet about the inside of the sample such as the chain force network, displacement vectors, etc.

We also conducted simulations with realistic soil granules. The results showed that Bullet can successfully capture the critical-state-type response of the soil. The present study therefore proved that Bullet can be used to study granular systems. However, it is important to note that, as in DEM simulations, the number of the simulated particles is limited by the amount of memory available in the system.

The present study was a part of an ongoing research effort looking at the applicability of physics engines to the simulation of geotechnical engineering problems. Further simulations to determine the accuracy of the Bullet physics engine for coupled analysis when rigid body systems come in contact with soft bodies as well as fluids are planned for the future.

## Supporting information

S1 File Geo-Bullet source code: “S1_File.zip”. (ZIP)
