# Supplementary figures and images for: Simulating direct shear tests with the Bullet physics library: A validation study
Source: PLoS One. 2018 Apr 19;13(4):e0195073. doi: 10.1371/journal.pone.0195073 (PMC5908395; doi:10.1371/journal.pone.0195073)

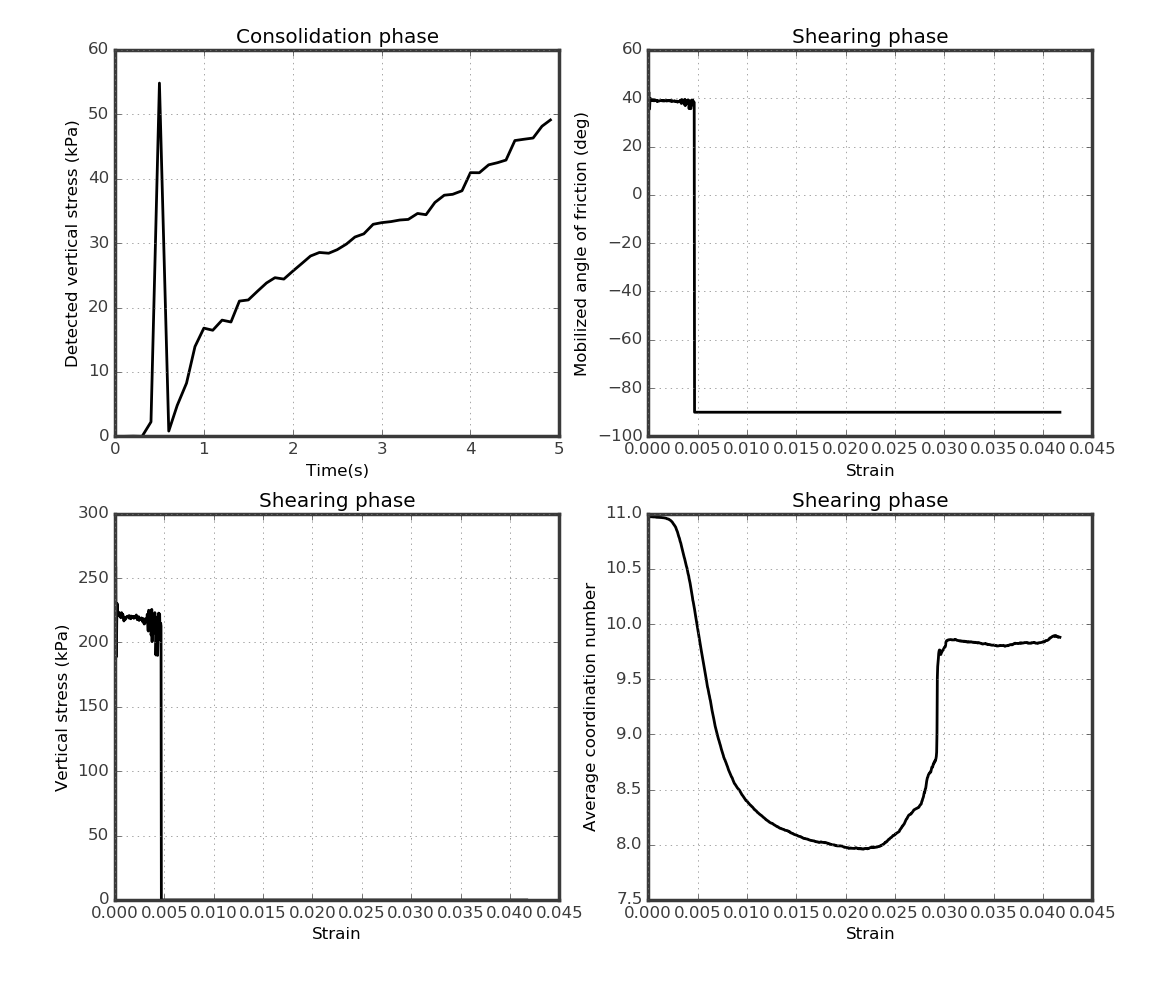

Supplement: S1 File — (ZIP) [file pone.0195073.s001.zip › Geo-Bullet-master/triaxialOnBeads/figure_1.png]
